# Dysfunction in Ankyrin-B Disrupts Heart Rate Regulation by Altering the Membrane Abundance of GIRK Channels

**DOI:** 10.1016/j.jacbts.2026.101647

**Published:** 2026-08-11

**Authors:** Jingdong Li, Crystal F. Kline, Omer Cavus, John T. Bobik, Nipun Malhotra, Jagun Raghavan, Sarah S. Takenaka, Junie Kwak, Ryan J. Cordeiro, Eleanor J. Mohler, Emma Dudley, Xianyao Xu, Drew M. Nassal, Yuejin Wu, Thomas J. Hund, Matteo E. Mangoni, Peter J. Mohler, Mona El Refaey

**Affiliations:** a The Frick Center for Heart Failure and Arrhythmia, Dorothy M. Davis Heart and Lung Research Institute, The Ohio State University, Columbus, Ohio, USA; b Division of Cardiac Surgery, Department of Surgery, The Ohio State University College of Medicine and Wexner Medical Center, Columbus, Ohio, USA; c Physiology and Cell Biology, The Ohio State University College of Medicine and Wexner Medical Center, Columbus, Ohio, USA; d Department of Medicine, Johns Hopkins University, Baltimore, Maryland, USA; e Department of Biomedical Engineering, College of Engineering, The Ohio State University, Columbus, Ohio, USA; f Division of Cardiovascular Medicine, Department of Internal Medicine, The Ohio State University College of Medicine and Wexner Medical Center, Columbus, Ohio, USA; g Institut de Génomique Fonctionnelle, Université de Montpellier, CNRS, INSERM, Montpellier, France; h Laboratories of Excellence, Ion Channel Science and Therapeutics, Valbonne, France

**Keywords:** ankyrin-B, autonomic nervous system, *I*_K,ACh_, *I*_Ca,L_, GIRK1, GIRK4, SAN, desensitization

## Abstract

Ankyrin-B (AnkB), encoded by *ANK2*, is essential for ion channel localization in the heart. We investigated its role in regulating sinoatrial node (SAN) responses to autonomic input. AnkB-haploinsufficient (AnkB^+^/^−^) mice showed baseline bradycardia, heightened SAN response to β-adrenergic stimulation, and increased heart rate variability. These mice exhibited reduced *I*_Ca,L_ and SAN sarcoplasmic reticulum Ca^2+^ content, unchanged *I*_K,ACh_ density, but impaired *I*_K,ACh_ desensitization. AnkB interacts with GIRK1/4 subunits, and its loss in cardiomyocyte-specific knockout mice reduced GIRK1 membrane localization. Our findings support that AnkB deficiency disrupts autonomic regulation of SAN function through downregulation of *I*_Ca,L_ and the reduction in *I*_K,ACh_ desensitization.

## Highlights

• AnkB^+/-^ mice display bradycardia and heightened response to β-adrenergic stimulation.
• AnkB^+/-^ mice maintain unchanged *I*_K,ACh_ density but impaired *I*_K,ACh_ desensitization.
• AnkB^+/-^ mice exhibit reduced atrial expression of GIRK1 with no change in GIRK4.
• *Ank2-*cKO mice show reduced atrial GIRK1 membrane localization but unchanged GIRK4.

## Visual Abstract

**Figure:**
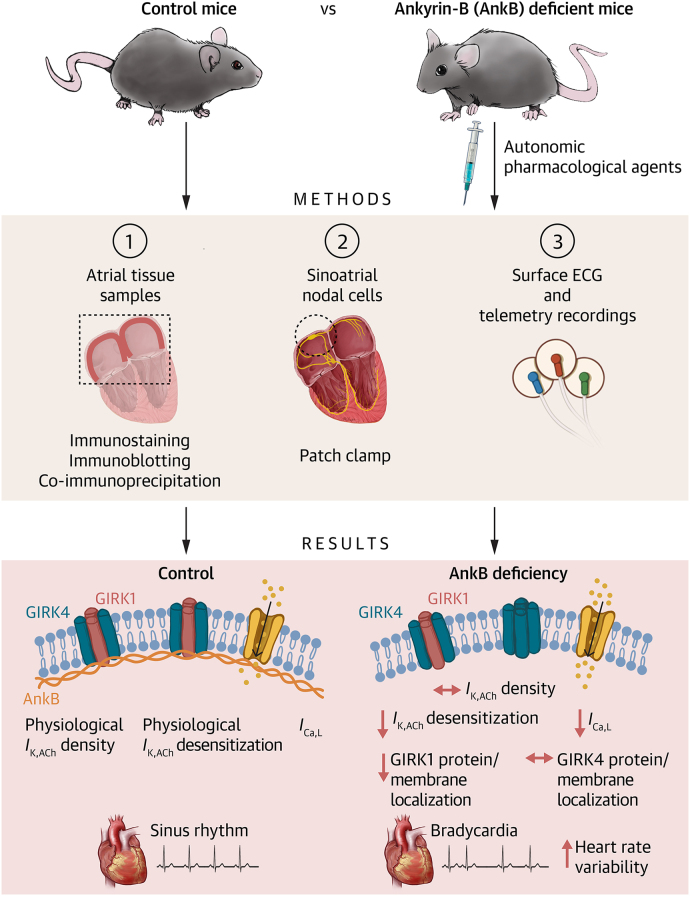

Work from our group and others supports atypical mechanisms of arrhythmia due to dysfunction in proteins that are required for proper expression and local regulation of ion channels and transporters at specific excitable membranes.^1^^,^^2^ Ankyrin-B (AnkB), encoded by *ANK2*, is a scaffolding protein that organizes critical ion channels and transporters at the membrane of cardiomyocytes. Disruption of AnkB function or expression impairs cardiac excitability and elevates the risk of arrhythmias.^3^ Mice heterozygous for AnkB deficiency have shown cardiac phenotype consisting of bradycardia, sinus node dysfunction, heart rate (HR) variability, and increased risk of sudden cardiac death associated with adrenergic stimulation.^1^^,^^2^^,^^4^ Previous work highlighted a role of AnkB in the regulation of Ca_v_1.3 membrane targeting and function in atrial myocytes^5^ and sinoatrial node (SAN) cells.^4^ Specifically, earlier studies have shown a reduction in the membrane distribution of Ca_v_1.3 in AnkB^+/–^ SAN cells in contrast to the continuous membrane distribution in WT cells.^4^ Additionally, cardiomyocyte-specific loss of AnkB expression (*Ank2*-cKO) in mice was linked to ventricular chamber remodeling, widespread fibrosis, and atrial enlargement.^6^

*KCNJ3* encodes the G protein–coupled inwardly rectifier potassium channel subtype 1 protein (GIRK1/Kir3.1), and *KCNJ5* encodes the G protein–coupled inwardly rectifier potassium channel subtype 4 protein (GIRK4/Kir3.4). GIRK1 and GIRK4 are the predominant *GIRK* gene products in the heart and assemble as tetramers to regulate acetylcholine/adenosine-induced potassium current (*I*_K,ACh_).^7^^,^^8^ Moreover, GIRK1 and GIRK4 are mainly expressed in the SAN, atrioventricular node (AVN), and atria.^9^^,^^10^ While GIRK4 subunits predominantly support K^+^ flux, GIRK1 subunits have significant regulatory impact on K_ACh_ channel gating, conductance, and ATP modulation.^11^, ^12^, ^13^, ^14^ Because of their key role in autonomic regulation of HR and SAN pacemaker activity,^12^^,^^15^ we hypothesized that GIRK channels may represent an unidentified effector of *ANK2* loss-of-function phenotype.

*Ank2*-haploinsufficient (AnkB^+/−^) mice displayed increased HR response to the β-adrenergic agonist (isoproterenol [ISO]) and the muscarinic receptor agonist (carbachol [CCH]) stimulation. These mice also showed an increase in heart rate variability (HRV) parameters at baseline compared to control animals. We also report a decrease in *I*_Ca,L_ density and a reduction in SAN SR Ca^2+^ content in AnkB^+/−^ SAN cells along with reduced *I*_K,ACh_ desensitization. Our data demonstrate a novel role of AnkB in the sympathetic/parasympathetic modulation of HR, via the expression of the GIRK1 subunit at the membrane. At the cell and molecular level, we identify AnkB as cardiac binding partners of GIRK subunits and illustrate altered GIRK channel membrane localization and *I*_K,ACh_ channel regulation in myocytes with reduced AnkB expression.

## Methods

### Data availability

All data are contained within the article and supporting document.

### Animals

All animal procedures were approved and are in accordance with institutional guidelines. Mice used were age- and sex-matched, were housed in the same facility (temperature and humidity), consumed the same diet, and were kept on identical 12-hour light/dark cycles. AnkB^+/−^ mice^16^ were backcrossed into C57BL/6 background (Jackson Laboratories) before experiments. *Ank2*-cKO mice (cardiomyocyte specific) were produced as previously described.^6^ Mice were also backcrossed onto a C57BL/6 background. Animals were crossed with mice expressing the Cre-recombinase under the cardiac αMHC, to induce the specific loss of AnkB in adult cardiac myocytes.

### Tissue preparation

For immunoblotting and co-immunoprecipitation analysis, atria or total heart tissues were harvested, washed in ice-cold phosphate-buffered saline (PBS), and flash-frozen in liquid nitrogen. The frozen tissues were resuspended in ice-cold homogenization buffer containing 1× protease inhibitor cocktail (Thermo Fisher 78429) and phenylmethylsulfonyl fluoride (PMSF; Sigma P7626). The tissue was homogenized using Precellys 24 Touch bead homogenizer at 6× (6,000 rpm for 15 seconds), followed by centrifugation at 4 °C for 45 minutes. The resulting supernatant was quantified by bicinchoninic acid assay (BCA; Thermo-Scientific) before analysis.

### Reagents

Anti-ankyrin-B Ig (custom antibody produced by Covance),^6^ anti-GIRK1 (Alomone Labs), anti-GIRK4 (Alomone Labs), and anti-GAPDH (Fitzgerald Industries Inc).

### Co-immunoprecipitations

To ensure specificity in co-immunoprecipitation experiments, samples including membrane fractions were fully homogenized. Samples were then subjected to ultracentrifugation, and only the resulting supernatant was used for co-immunoprecipitation experiments, as we have previously described.^6^ First, for immunoblotting with anti-AnkB, around 600 and 800 μg of atrial and total heart lysates supernatant were incubated with control IgG, anti-GIRK1 Ig, or anti-GIRK4 Ig on a rotator together for 12 to 16 hours at 4 °C. After incubation, Protein A/G Magnetic Beads (Pierce, No. 88802) were added and kept on a rotator at 4 °C for 4 to 5 hours. A magnetic stand was used to remove the supernatant and washed 3 times with PBS including 500 mM NaCl. Second, for immunoblotting with anti-GIRK1 and anti-GIRK4, around 400 μg of atrial lysate and 1,000 μg of total heart lysate were incubated with anti-ankyrin-B Ig bound beads (using Pierce Co-Immunoprecipitation Kit, Thermo 26149) on a rotator for 12 to 16 hours at 4 °C. After washing in both protocols, (2× or 4×) Laemmli sample buffer with β-mercaptoethanol was added to elute the protein, then boiled to 95 °C for 5 minutes. The proteins were separated by SDS-PAGE before immunoblotting with anti-ankyrin-B Ig, anti-GIRK1 Ig, or anti-GIRK4 Ig.

### Immunoblotting

Around 40 μg of atrial lysate was used and run on either 4% to 15% precast ProteanTGX gels (Bio-Rad 4561084) or any kD precast ProteanTGX gels (Bio-Rad 4568124). The gel was then transferred onto a nitrocellulose membrane (Bio-Rad). The membrane was blocked for 1 hour at room temperature in casein (Bio-Rad 1610782 or Thermo Fisher 37532) and then incubated in primary antibody at 4 °C overnight. Primary antibodies used targeted ankyrin-B (Covance, 1:1,000),^6^ anti-K_ir_ 3.1 (GIRK1, 1:1,000; Alomone Labs APC-005), anti-K_ir_ 3.4 (GIRK4, 1:1,000; Alomone Labs APC-027), anti-RGS4 (D4V1P) (RGS4, 1:1,000; Cell Signaling 15129), anti-RGS6 [EPR6342] (RGS6, 1:1,000; Abcam ab128943), and anti-GAPDH (1:5,000; Fitzgerald 10R-G109a). After washing, the membrane was put in secondary antibodies donkey antirabbit (1:7,500; Jackson Immuno-Research Laboratories 711-035-152) and donkey antimouse (1:7,500; Jackson Immuno-Research Laboratories 715-035-150) for 2 hours at room temperature. Membranes were briefly washed in Clarity western ECL (Bio-Rad 170-5061) or Super-Signal West Femto (Thermo Scientific 34096) enhanced chemiluminescent substrate before imaging. Membrane was imaged on ChemiDoc MP Imager and quantified using ImageJ software.

### Immunostaining of heart sections

Whole heart samples from *Ank2*^fl/fl^ and *Ank2*-cKO mice were dissected following euthanasia. Whole hearts were washed in PBS (Thermo 10010049), embedded in OCT (VWR 25608-930), and snapped frozen in liquid nitrogen vapors. For cryoslices, 4-chamber heart sections from *Ank2*^fl/fl^ and *Ank2*-cKO mice were cut at 10-μm thickness and blocked for 1 hour in blocking solution (3% fish gelatin, 1% DMSO, 0.75% Triton X-100 (10%), in 1× PBS). Sections were incubated overnight with primary antibodies targeting anti-K_ir_ 3.1 (GIRK1, 1:100; Alomone Labs APC-005) and anti-K_ir_3.4 (GIRK4, 1:50; Alomone Labs APC-027) at 4 °C. After overnight incubation, secondary antibodies, donkey antirabbit 488 (Thermo A21206), and Wheat Germ Agglutinin CF 568 (WGA, 1:400; Biotium 29077-1) were used to incubate the sections for 2 hours at room temperature. After incubation, the sections were extensively washed with blocking solution and covered with DAPI-containing Vectashield imaging medium (Vector Laboratories), and coverslips (No. 1) were applied. Images were acquired with a confocal microscope using 20× acquisition. Images were collected using similar confocal protocols at room temperature, with the observer blinded to the genotype. Secondary antibodies were used as negative controls and imaged at the same gain.^6^^,^^17^ Images were quantified using ImageJ software.

### Telemetry implantation and ECG recording

Conscious electrocardiograms (ECGs) were collected using implanted radio telemetry devices using an ETA-F10 miniature telemeter (DSI) and Dataquest A.R.T acquisition software (https://www.datasci.com/products/software/ponemah) for HRV measurements.

### Fast Fourier transformation analysis

Fast Fourier transformation (FFT) analysis was used to analyze HRV by converting time domain into frequency domain. ECGs were recorded on LabChart software (ADInstruments), and noise was manually removed. RR intervals were further screened for noise using the arithmetic function on LabChart (ADInstruments), then exported into 2-minute increment text files made in Notepad. The text files containing the raw RR intervals were FFT analyzed via Custom MATLAB code^18^ and used to determine the spectral frequency (low frequency, 0.15-1.5 Hz and high frequency, 1.5-5 Hz), standard deviation of intervals between the consecutive heart beats (SDRR), instantaneous beat-to-beat interval variability (SD1), and continuous long-term R-R interval variability (SD2) values.

### Surface ECG recording

Surface ECG was recorded in mice that were kept at a temperature of 35 °C and anesthetized by isoflurane (∼1%) inhalation using subcutaneous electrodes. After 10 minutes of baseline recordings, pharmacological compounds (0.4 mg/kg ISO, 0.1 mg/kg CCH, 1 mg/kg atropine and 1 mg/kg propranolol) to activate or block the autonomic nervous system were delivered by intraperitoneal injection. A constant level of anesthetic was maintained throughout the recording period.

### Electrophysiology in Langendorff-perfused hearts

These procedures were performed using a protocol modified from Khoo et al.^19^ Briefly, AnkB^+/−^ and WT control mice were anesthetized, and surface ECG was recorded continuously for 2 minutes. Next, the heart was excised and rinsed in iced Tyrode solution containing (in mM) 137 NaCl, 5.4 KCl, 0.5 MgCl_2_, 0.16 NaH_2_PO_4_, 3 NaHCO_3_, 5 HEPES-NaOH, and heparin 1 mg/10 mL, adjusted to pH 7.4 with NaOH at room temperature. Then, the heart was immediately perfused retrogradely through the aorta for 1 to 2 minutes at room temperature with Hanks balanced salt solution (Invitrogen-Gibco). The heart was then mounted on a modified Langendorff apparatus (HSE-HA perfusion system, Harvard Apparatus Inc) for retrograde aortic perfusion at a constant pressure of 80 mm Hg with carbogen (95% O_2_, 5% CO_2_) Krebs-Henseleit bicarbonate solution consisting of (in mM) 25 NaHCO_3_, 118 NaCl, 4.7 KCl, 1.2 MgSO_4_, 1.2 NaH_2_PO_4_, 2.5 CaCl_2_, 0.5 Na-EDTA, and 15 glucose, with pH equilibrated to 7.4. The perfused heart was then immersed in the water-jacketed bath and maintained at 36 °C. After the heart was allowed to stabilize for 10 minutes, baseline Langendorff-perfused heart ECG recordings were obtained. Sinus node recovery time (SNRT) was assessed by right atrial pacing for 30 seconds at a cycle length of 100 ms and measured as the time from the last-paced beat to the first sinus beat. Right atrial stimulation was performed to assess the AV nodal Wenckebach interval, while a train of 8 beats at a cycle length of 100 ms (S1) followed by the delivery of a single premature beat (S2) were used to measure the AV nodal effective refractory period. Left ventricular stimulation with identical S1 and S2 was used to determine the left ventricular effective refractory period. The S2 interval was decremented by 1 ms in multiple runs to determine the AV nodal and left ventricular effective refractory periods.

### SAN cell isolation and electrophysiology

Single SAN cells were isolated from a protocol modified from DiFrancesco et al^20^ and Mangoni and Nargeot.^21^ Briefly, AnkB^+/−^ and WT control hearts were excised and placed into Tyrode solution (4 °C), consisting of (mM) 140 NaCl, 5.0 HEPES, 5.5 glucose, 5.4 KCl, 1.8 CaCl_2_, and 1.0 MgCl_2_, with pH adjusted to 7.4 with NaOH. The SAN region, delimited by the crista terminalis, atrial septum, and orifice of superior vena cava, was dissected from the heart. The SAN was cut into small strips, which were transferred and rinsed in a “low Ca^2+^” solution (35 °C) containing (mM) 140 NaCl, 5.0 HEPES, 5.5 glucose, 5.4 KCl, 0.2 CaCl_2_, 0.5 MgCl_2_, 1.2 KH_2_PO_4_, 50 taurine, and 1 mg/mL BSA with pH adjusted to 6.9 with NaOH. SAN tissue pieces were digested in 5 mL of “low CaCl_2_” solution (35 °C) containing collagenase type I, elastase (Worthington), and protease type XIV (Sigma) for 20 to 30 minutes. Then, the tissues were transferred to 10 mL of Kraft-Bruhe medium (35 °C) containing (in mM) 100 potassium glutamate, 5.0 HEPES, 20 glucose, 25 KCl, 10 potassium aspartate, 2.0 MgSO_4_, 10 KH_2_PO_4_, 20 taurine, 5 creatine, 0.5 EGTA, and 1 mg/mL BSA, with pH adjusted to 7.2 with KOH. The tissues were agitated using a glass pipette for 10 minutes. SAN cells were identified by their characteristic morphology (spindle or spider shape) and spontaneous activity. SAN cells were identified electrophysiologically by typical spontaneous action potentials (APs) with slow depolarizing phase 4 and the hyperpolarization-activated current *I*_f_.

Spontaneous APs were recorded using the perforated (amphotericin B) patch-clamp technique on single SAN cells adapted from Wu et al^22^ and Cifelli et al^23^ at 35 ± 1 °C in Tyrode solution containing (in mM) 140 NaCl, 5 KCl, 1 MgCl_2_, 1 CaCl_2_, 10 HEPES, and 5 glucose, with pH adjusted to 7.4 with NaOH. The pipette solution contained (in mM) 135 KCl, 0.1 CaCl_2_, 1 MgCl_2_, 5 NaCl, 10 EGTA, 4 Mg-ATP, 6.6 Na-phosphocreatine, 0.3 Na-GTP and 10 HEPES, with pH adjusted to 7.2 with KOH. Amphotericin B (200 μg/mL) was added to this pipette solution to record APs with the perforated patch-clamp technique. SAN cells with stable APs were included in the experiments. The effects of ISO dose-response and CCH dose-response on spontaneous AP frequency were investigated.

*I*_f_ was recorded using the whole-cell patch-clamp technique at 35 ± 1 °C using a protocol adapted from Cifelli et al^23^ and Cho et al.^24^ Pipette and extracellular solution are the same as in APs recording. After the initial current recordings, 0.5 mM BaCl_2_ was applied to extract the Ba^2+^-sensitive potassium component. The conditioning voltage steps were applied for 3.0 seconds ranging from −120 mV to −40 mV in 10-mV increments.

*I*_K,ACh_ was recorded at room temperature using the same pipette and extracellular solution as in APs and *I*_f_ recording.^23^ The *I*_K,ACh_ current-voltage (I-V) relationship was investigated using a series of voltage steps in 10-mV increments between −120 and 50 mV from a holding potential of −75 mV before and after the application of 10 μM CCH. The CCH-sensitive difference currents were assessed as described.^23^ *I*_K,ACh_ desensitization was measured by applying a long −100 mV voltage step (holding potential was −75 mV) after treating for 2 minutes with 10 μM CCH. *I*_K,ACh_ desensitization was calculated as the percentage of the peak current using the formula (*I*_1_ − *I*_2_)/*I*_1_ × 100. Here, *I*_1_ represents the peak current divided by cell membrane capacitance (C_m_). *I*_2_ represents the current at the end of recording divided by C_m_.

*I*_Ca,L_ was measured at room temperature using protocol adapted from Yeh et al.^25^ The external solution for *I*_Ca_ recording contained (in mM) 136 tetraethylammonium chloride, 5.4 CsCl, 2 CaCl_2_, 0.8 MgCl_2_, 10 HEPES, and 10 dextrose with pH adjusted to 7.4 with CsOH. Niflumic acid (50 μM) was added to inhibit Ca^2+^-dependent Cl-current. The internal solution for *I*_Ca_ recording contained (in mM) 120 CsCl, 20 TEA-Cl, 1 MgCl_2_, 5 Mg-ATP, 0.1 Li-GTP, 10 EGTA, and 10 HEPES with pH adjusted to 7.3 with CsOH.

### SR Ca ^2+^ content measurements

The SR Ca^2+^ content of SAN cells was measured by integrating the Na^+^/Ca^2+^ exchanger current (*I*_NCX_) in response to a “spritz” of caffeine using a protocol adapted from Wu et al.^22^ Briefly, cells were held at −80 mV for >5 minutes with pipette solutions. Cells were loaded with Ca^2+^ by stepping the cell membrane from −80 to 0 mV with a duration of 300 ms at 0.5 Hz (36 ± 1 °C). The bath solution contained (in mM): 137 NaCl, 10 HEPES, 10 glucose, 1.8 CaCl_2_, 0.5 MgCl_2_, and 25 CsCl with pH adjusted to 7.4 with NaOH. The intracellular pipette solution contained (in mM): 120 CsCl, 10 TEA, 1.0 Mg-ATP, 1.0 Na-GTP, 5.0 phosphocreatine, 10 HEPES, and Indo-1 0.2, with pH adjusted to 7.2 with 1.0 N CsOH. Voltage-activated Na^+^ current was inactivated by a 50-ms depolarization step to −40 mV before completing the depolarization to 0 mV. The K^+^ currents were eliminated by adding Cs^+^ and TEA and omitting K^+^ from the pipette and bath solutions. After loading Ca_i_^2+^ with >15 conditioning command steps, 20 mM caffeine was locally applied by a pipette close to the cell of study (DAD-12 Superfusion System, ALA Scientific Instruments) while the cell was holding at −80 mV. The *I*_NCX_ currents induced by caffeine were integrated and normalized to cell size.

### Biotinylation

An in vivo biotinylation protocol of the hearts of *Ank2*^fl/fl^ and *Ank2*-cKO mice was adapted from Rybak et al.^26^ Using a gravity-based perfusion setup with 25-mL syringes, hearts were perfused by puncturing the left ventricle with a small needle (25-gauge butterfly cannula). The right atria were also punctured to allow flow through of the perfusion solutions. The hearts were perfused with 3 different solutions depending on the experimental condition. About 15 to 20 mL was used per solution per mouse.

Solution #1 (same for all animals): Sterile PBS with 10% weight/volume Dextran-40 (TCI Chemicals) prewarmed to 38 °C and perfused for 10 minutes.

Solution #2A (used for nonbiotin control): Same as solution #1, prewarmed and perfused for 10 minutes.

Solution #2B (contains biotin): Sterile PBS with 1 mg/mL sulfo-NHS-LC-biotin (Apexbio), 10% wt/vol Dextran-40, prewarmed and perfused for 10 minutes.

Solution #3 (same for all animals): Prewarmed PBS with 10% Dextran-40 and 50 mM Tris for neutralization, perfused for 10 minutes.

The tissues were dissected and snapped frozen in liquid nitrogen and stored at −80 °C.

Samples were homogenized using the Precellys 24-bead homogenizer using harsh lysis buffer (400 μL/atrial sample) containing 1 mL 0.1 M NaPO_4,_ pH 6.8; 8 μL 0.25 M EDTA; 100 μL 1 M NaN_3_; 0.07 g NaCl; 1 mL 10% SDS; 100 μL BME, then brought to total volume of 10 mL, and protease inhibitors were added just before use. Protein concentration was calculated using the RC/DC protein assay.

A small fraction of lysate was retained for preparation of input control. Streptavidin-Sepharose beads (BioVision) were spun 3× at 1,800*g* for 5 minutes at 4 °C, then resuspended in PBS (approximately 60 μL beads for every 400 μg of protein, brought to total volume of 100 μL with PBS). The bead and protein mixture were incubated overnight at 4 °C with end-over-end rotation. The following day, bead slurries were spun at 1,800*g* for 3 minutes at 4 °C; supernatant was removed and saved for flow-through. Beads were washed with PBS + 500 mM NaCl, PBS + 1M NaCl, and PBS (about 20 μL) under same conditions with supernatant discarded between each spin. A 4× Laemmli buffer with BME was added (1:1 [vol/vol]) to beads. Flow-through fraction and input lysate were prepared. The beads were boiled at 95 °C for 25 minutes and spun at maximum speed for 3 minutes. Immunoblotting was performed as previously described. Primary antibodies included GIRK1 (1:500 in casein), Streptavidin-biotin Dylight (1:1,200; Bio-Rad STAR152D800GA). The blots were washed the following day and incubated with Starbright 520 (Bio-Rad 12005869; 1:1,500 in casein) secondary antibody for at least 1 hour. Blots were imaged at 520 and 800 nm and quantified using ImageJ.

### Statistics

Data are presented as mean ± SEM. Two independent groups were evaluated for normality using the Shapiro-Wilk test on GraphPad Prism (version 10.3.1 for Windows, GraphPad Software). A *P* value of <0.05 was used to determine if data passed or failed the Shapiro-Wilk test. Two independent groups were compared using the unpaired Student's *t-*test if data passed the Shapiro-Wilk test or the Mann-Whitney *U* test if data failed the Shapiro-Wilk test. Two groups with 2 variables were first screened on GraphPad Prism for model residuals, homoscedasticity, and QQ plots. Then, data were analyzed using 2-way analysis of variance (ANOVA) with repeated measures (RM). The time course of pharmacological agonist(s)/antagonist(s) and the drug dose were considered the within-subject factors for the RM ANOVA. All mice that were analyzed were exposed to same time course or drug dose. Sphericity was not assumed, so the Geisser-Greenhouse correction was applied. To control for false discovery rate, the Benjamini, Krieger, and Yekutieli's 2-stage linear step-up procedure for multiple comparisons was used. Comparisons that contained psuedoreplicates were evaluated using a linear mixed-effects model (LMM) in RStudio (version 4.6.0, Posit Software). Models generated using LMM were evaluated for normality using the Shapiro test on RStudio. Data that passed Shapiro test were analyzed using LMM, while data that failed were analyzed using generalized linear mixed effects model (GLMM) in RStudio. To control for false discovery rate, Benjamini-Hochberg procedure for multiple comparisons was performed. For all experiments, the unit of analysis was the mouse. A 2-sided *P* value of <0.05 was considered statistically significant.

## Results

### AnkB deficiency alters HR response to sympathetic/parasympathetic agonists

We first tested the role of AnkB in the regulation of HR. Mice globally heterozygous for *Ank2* null mutation (*Ank2* haploinsufficient knockout mice, AnkB^+/−^) displayed reduced basal HR compared to WT controls at baseline (Figures 1A to 1D; ∗*P* < 0.05). Injection of ISO abolished these differences in AnkB^+/−^ mice because of a greater net increase in HR in AnkB^+/−^ mice (Figures 1A to 1D; ∗*P* < 0.05, ∗∗*P* < 0.01). To investigate how *Ank2* deficiency alters HR response to the activity of sympathetic and parasympathetic branches of the autonomic nervous system, HRV analysis was performed in conscious mice.^18^ The standard deviation of intervals between the consecutive heart beats (SDRR), the percentage of successive RR interval pairs that differ by more than 6 milliseconds (pNNper6ms), instantaneous beat-to-beat interval variability (SD1), continuous long-term R-R interval variability (SD2), and spectral frequency (low frequency [LF], 0.15-1.5 Hz and high frequency [HF], 1.5-5 Hz) were all determined by fast Fourier transformation analysis.^18^ Of note, the HF component is modulated by the parasympathetic nervous system and the LF component reflects the activity of both sympathetic and parasympathetic nervous system.^27^ We observed a significant increase in all parameters including SDRR, pNNper6ms, SD1, SD2, LF, and HF in AnkB^+/−^ mice at baseline compared to control mice (Supplemental Figure 1A; ∗*P* < 0.05, ∗∗*P* < 0.01). Injection of ISO abolished these differences in AnkB^+/−^ mice and showed no significant changes across all parameters (Supplemental Figure 1B).

**Figure 1:**
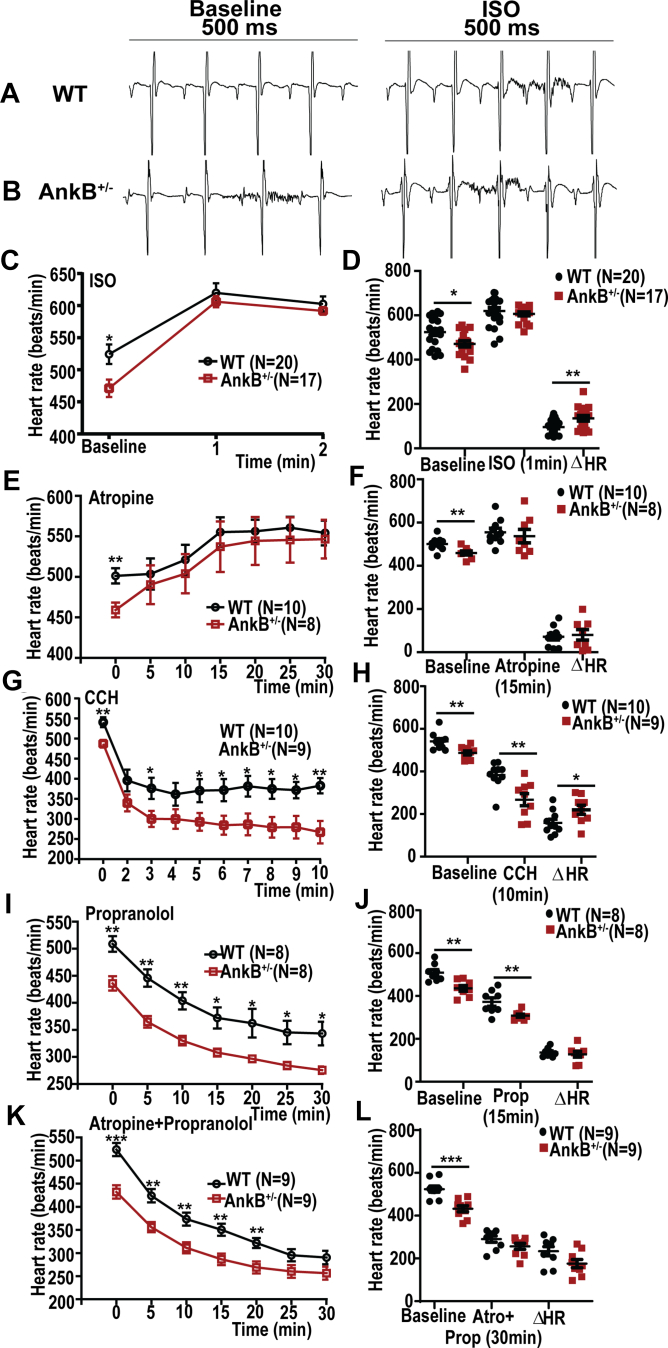
Impact of AnkB Deficiency on Heart Rate Response to Sympathetic/Parasympathetic Agonists (A and B) Representative surface ECG recordings from WT and AnkB^+/−^ mice at baseline and after ISO injection. AnkB^+/−^ mice display reduced HR compared to WT controls (WT: n = 20 mice, AnkB^+/−^: n = 17 mice). (C and D) Injection of ISO normalizes the differences in HR given a great net increase in HR in AnkB^+/−^ mice (WT: n = 20 mice, AnkB^+/−^: n = 17 mice). (E and F) Injection of atropine abolishes the HR differences between AnkB^+/−^ mice and their WT controls (WT: n = 10 mice, AnkB^+/−^: n = 8 mice). (G and H) Injection of CCH further reduces HR in AnkB^+/−^ mice (WT: n = 10 mice, AnkB^+/−^: n = 9 mice). (I and J) Injection of propranolol reduces resting HR equivalently in both genotypes (WT: n = 8 mice, AnkB^+/−^: n = 8 mice). (K and L) Injection of propranolol plus atropine results in equivalent reductions at early time points but not in later time points in AnkB^+/−^ mice (WT: n = 9 mice, AnkB^+/−^: n = 9 mice). Drug doses: 0.4 mg/kg ISO, 1 mg/kg atropine, 0.1 mg/kg CCH, 1 mg/kg propranolol. ΔHR denotes magnitude of HR change. Data are presented as mean ± SEM. Data were analyzed using unpaired Student's *t*-test (D: baseline and ISO (1 minute); F, H: Baseline and ΔHR, J, L), Mann-Whitney *U* test (D: ΔHR and H: CCH [10 minutes]), and 2-way repeated measures ANOVA with Benjamini, Krieger and Yekutieli's 2-stage linear step-up procedure for multiple comparisons (C, E, G, I, K); ∗*P* < 0.05, ∗∗*P* < 0.01, ∗∗∗*P* < 0.001. CCH = carbachol; ECG = electrocardiogram; HR = heart rate; ISO = isoproterenol.

We then tested HR response in the presence of parasympathetic blockade using atropine, a potent muscarinic receptor antagonist. Atropine had minimal effects on HR in control animals by 5 minutes and induced a similar increase in HR in both genotypes at 15 minutes post-administration (Figures 1E and 1F; ∗∗*P* < 0.01). However, administration of CCH further attenuated the already reduced HR in AnkB^+/−^ mice compared with control animals (Figures 1G and 1H; ∗*P* < 0.05, ∗∗*P* < 0.01). Injection of CCH also eliminated the differences in HRV parameters across genotypes observed at baseline (Supplemental Figure 1C).

Injection of the β-adrenergic receptor blocker (propranolol) reduced resting HR equivalently across genotypes at all times examined (Figures 1I and 1J; ∗*P* < 0.05, ∗∗*P* < 0.01). We also investigated HR regulation to simultaneous sympathetic and parasympathetic blockers by examining HR response to propranolol plus atropine. Propranolol plus atropine resulted in equivalent reductions in HR at 5, 10, 15, and 20 minutes in AnkB^+/−^ mice vs control mice (Figures 1K and 1L; ∗∗*P* < 0.01, ∗∗∗*P* < 0.001). Reduced heart rates in AnkB^+/−^ mice were noticeable at baseline, suggesting an exaggerated parasympathetic control of HR in AnkB^+/−^ mice. HRV parameters were also higher in AnkB^+/−^ mice at baseline compared to post-ISO stimulation, likely because of increase in heart rate along with sympathetic overdrive and reduced parasympathetic activity.

### AnkB dysfunction enhances HR response to sympathetic agonist signaling

To eliminate influences or variable response to anesthesia, we investigated HR response to ISO in isolated Langendorff-perfused hearts and isolated single SAN cells. Excised AnkB^+/−^ hearts showed a reduced HR compared with WT (Figures 2A to 2C; ∗∗*P* < 0.01, ∗∗∗*P* < 0.001). ISO elevated HR in both genotypes to equivalent levels given a greater net increase in AnkB^+/−^ vs WT HR (Figures 2A to 2C; ∗∗*P* < 0.01, ∗∗∗*P* < 0.001). At the level of the single SAN cell, we observed reduced automaticity in the AnkB^+/−^ SAN cells compared with SAN cells from WT controls (Figures 2D and 2E; ∗*P* < 0.05). Similar to heart data, the enhanced positive chronotropic responses to ISO were observed in AnkB^+/−^ SAN cells that showed greater sensitivity to ISO-mediated increase in the SAN firing rates in AnkB^+/−^ than in WT SAN cells (Figures 2D to 2F, at 50 nM ISO; ∗*P* < 0.05, ∗∗*P* < 0.01). In summary, AnkB deficiency leads to intrinsic enhancement of sympathetic signaling, as evidenced by increased net response to ISO stimulation in isolated hearts and SAN cells.

**Figure 2:**
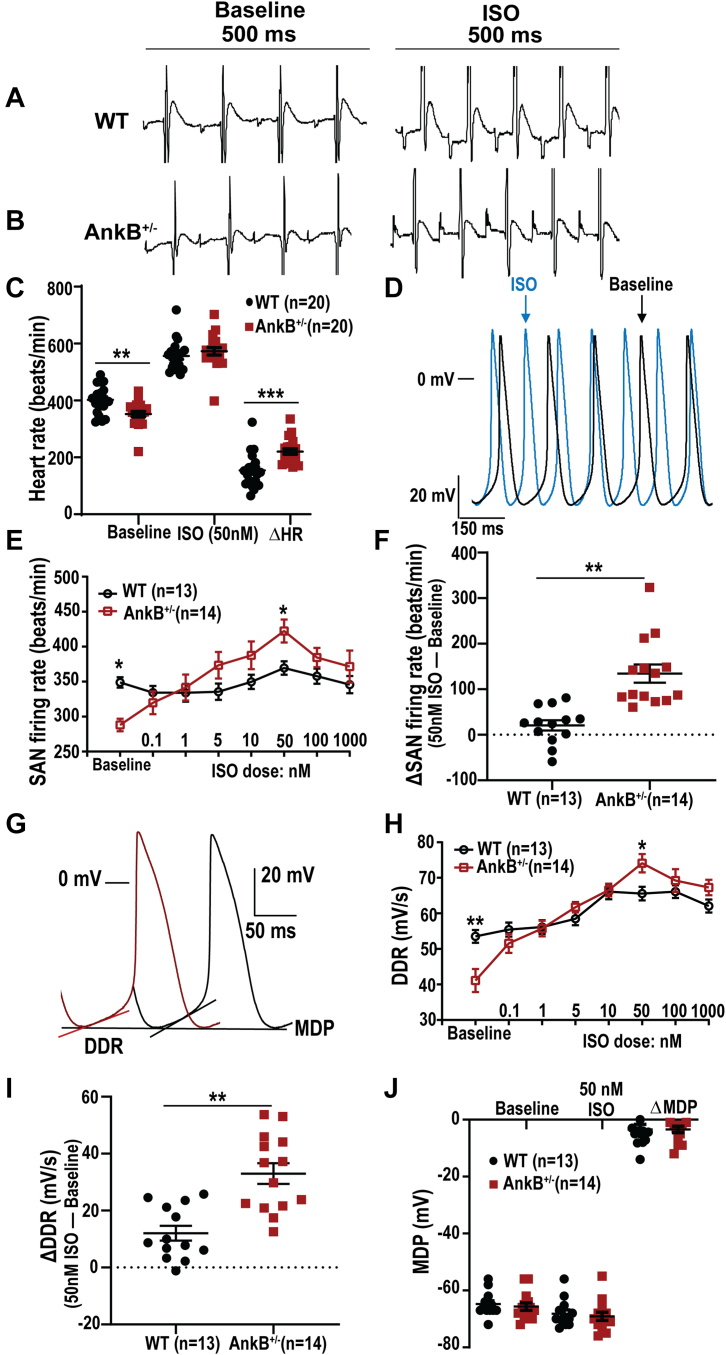
AnkB Deficiency Enhances Heart Rate Response to Sympathetic Signaling (A and B) Representative recordings from isolated hearts from AnkB^+/−^ mice showing a reduction in HR compared to WT (WT: n = 20 mice, AnkB^+/−^: n = 20 mice). (C) ISO treatment increases HR in AnkB^+/−^ mice to a level equivalent to control mice (WT: n = 20 mice, AnkB^+/−^: n = 20 mice). (D and E) AnkB^+/−^ SAN cells show an increase in SAN firing rates in response to ISO at 50 nM compared to WT cells (WT: n = 6 mice, n = 13 cells; AnkB^+/−^: n = 6 mice, n = 14 cells). (F) AnkB^+/−^ SAN cells show a higher net increase in SAN firing rates in response to 50 nM ISO compared to WT cells (WT: n = 6 mice, n = 13 cells; AnkB^+/−^: n = 6 mice, n = 14 cells). (G and H) SAN diastolic depolarization rate (DDR) is reduced in AnkB^+/−^ cells compared to WT cells. (H) DDR is increased after ISO stimulation at 50 nM in AnkB^+/−^ SAN cells compared to WT (WT: n = 6 mice, n = 13 cells, AnkB^+/−^: n = 6 mice, n = 14 cells). (I) AnkB^+/−^ SAN cells show a higher net increase in DDR in response to 50 nM ISO compared to WT cells (WT: n = 6 mice, n = 13 cells; AnkB^+/−^: n = 6 mice, n = 14 cells). (J) Maximum diastolic potential (MDP) is not different between WT and AnkB^+/−^ SAN at baseline and following ISO (WT: n = 6 mice, n = 13 cells; AnkB^+/−^: n = 6 mice, n = 14 cells). Data are presented as mean ± SEM. Data were analyzed using unpaired Student's *t*-test (C: ΔHR), Mann-Whitney *U* test (C: Baseline and ISO 50 nM), linear mixed-effects model with Benjamini and Hochberg's procedure for multiple comparisons (E, F, H, I, and J: ΔMDP) and generalized mixed-effects model with Benjamini-Hochberg's procedure for multiple comparisons (J: baseline and 50 nM ISO); ∗*P* < 0.05, ∗∗*P* < 0.01, ∗∗∗*P* < 0.001. HR = heart rate; ISO = isoproterenol; SAN = sinoatrial node.

### ISO stimulation upregulates diastolic depolarization rate in AnkB^+/−^ SAN cells

SAN cell diastolic depolarization rate (DDR), the slope of phase 4 depolarization, determines the rate of action potential firing.^28^ To further understand the cellular mechanisms underlying the altered response of SAN cell rate to sympathetic stimulation, we measured DDR with and without ISO stimulation. DDR showed a reduction in AnkB^+/−^ SAN cells at baseline (Figures 2G and 2H; ∗*P* < 0.05, ∗∗*P* < 0.01), consistent with reduced HR seen in AnkB deficiency in mice. Sympathetic stimulation increased DDR, leading to shortened diastolic intervals and faster SAN action potential (AP) triggering in SAN cells (Figures 2G and 2H; ∗*P* < 0.05, ∗∗*P* < 0.01). The DDR increased in accordance with increasing ISO, but DDR increases were significantly greater in AnkB^+/−^ cells compared with WT SAN cells at 50 nM ISO (Figures 2H and 2I; ∗*P* < 0.05, ∗∗*P* < 0.01), in line with observed increases in rate responsiveness of AnkB^+/−^ SAN cells to ISO. The maximum diastolic potential (MDP) was not different between WT and AnkB^+/−^ SAN cells (Figure 2J). No changes were noted in AP duration in AnkB^+/−^ SAN compared to WT cells at baseline and following ISO stimulation (Supplemental Figure 2).

### No changes are noted in SAN recovery time or AVN conduction in AnkB deficiency

QT_90_, QT_75_, and QT_50_ from surface ECGs were significantly prolonged in AnkB^+/−^ mice compared with WT controls (Supplemental Figure 3A; ∗∗*P* < 0.01). To obviate potential autonomic and anesthetic effects on SAN and AV conduction parameters, we studied the related parameters in Langendorff-perfused WT and AnkB^+/−^ hearts. AnkB^+/−^ hearts displayed significantly increased QT_90_, QT_75_, and QT_50_ compared with WT hearts (Supplemental Figure 3B; ∗*P* < 0.05). Unexpectedly, WT and AnkB^+/−^ hearts showed similar corrected sinus node recovery time (SNRTc), atrioventricular effective refractory periods (AV-ERP), AV nodal Wenckebach cycle length (AV-WBCL), and AV 2:1 conduction cycle length (Supplemental Figure 3C) values, suggesting that potential differences in AnkB^+/−^ hearts to ISO or CCH response were not likely to affect these electrophysiological parameters (Supplemental Figure 3C). Notably, left ventricular effective refractory periods (LV-ERPs) were significantly different between WT and AnkB^+/^ hearts (Supplemental Figure 3C; ∗∗∗*P* < 0.001).

### AnkB^+/−^ SAN exhibits enhanced parasympathetic/CCH response

Then, we evaluated the role of AnkB as a regulator of HR to parasympathetic stimulation using isolated hearts. HRs from Langendorff-perfused hearts of both genotypes were equivalently suppressed by a range of CCH concentrations (Figure 3A; ∗*P* < 0.05). However, to mimic the in vivo sympathetic and parasympathetic effects, as well as elucidate the difference of CCH response between in vivo and ex vivo experiments, a separate group of isolated hearts was treated with 50 nM ISO at all CCH concentrations (Figure 3B). Thus, the effects of increasing concentrations of the CCH were examined in ISO-stimulated hearts. With this approach, we demonstrated an increased CCH dose-response in AnkB^+/−^ hearts vs WT hearts (Figure 3B; ∗*P* < 0.05 at 0.5, 1, and 10 μM CCH and ∗∗*P* < 0.01 at 5 μM CCH). Thus, in the AnkB model, parasympathetic effects prevailed over the sympathetic effects, and AnkB dysfunction potentiates the SAN response to parasympathetic inhibition. In the presence of 50 nM ISO as a baseline condition, sinus arrest caused by CCH occurred earlier in AnkB^+/−^ hearts vs in WT hearts (Figure 3C; ∗*P* < 0.05).

**Figure 3:**
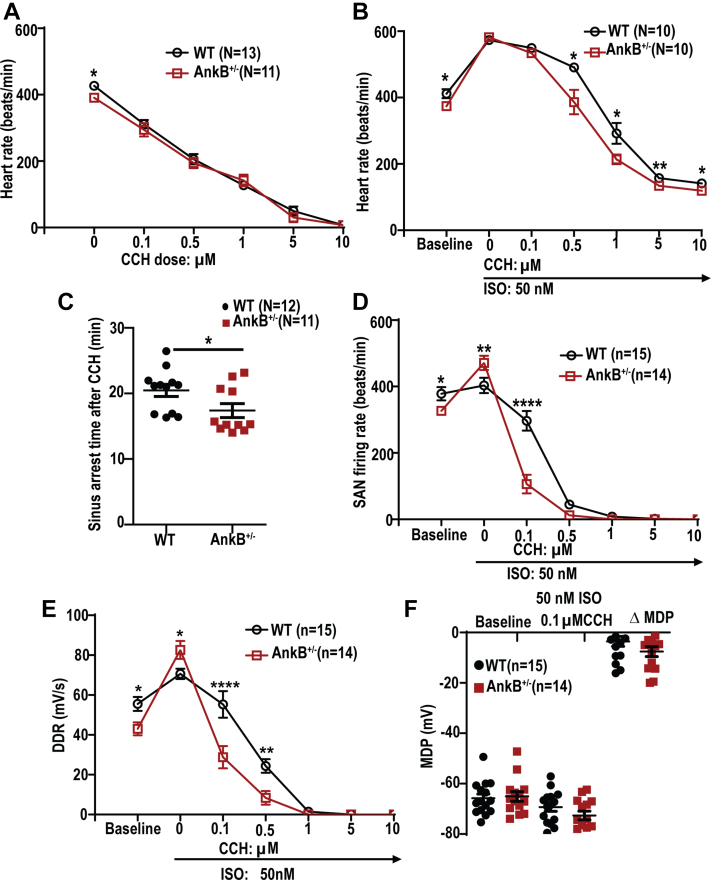
AnkB^+/−^ SAN Displays Enhanced Response to Parasympathetic Stimulation (A) HRs from Langendorff-perfused hearts of WT and AnkB^+/−^ are equivalently suppressed by CCH in concentrations ranging from 0.1 to 10 μM (WT: n = 13 mice, AnkB^+/−^: n = 11 mice). (B) ISO stimulation in AnkB^+/−^ hearts show an increased response to CCH treatment at 0.5, 1, 5, and 10 μM compared to WT hearts (WT: n = 10 mice, AnkB^+/−^: n = 10 mice). (C) In the presence of ISO baseline, sinus arrest in AnkB^+/−^ hearts occurs faster in response to CCH compared to WT controls (WT: n = 12 mice, AnkB^+/−^: n = 11 mice). (D) AnkB^+/−^ SAN cells show a reduced AP firing rate at baseline compared with WT SAN cells. 50 nM ISO increases firing rates in AnkB^+/−^ SAN cells. Coapplication of CCH significantly reduces the AP firing rate at 0.1 μM CCH (WT: n = 5 mice, n = 15 cells; AnkB^+/−^: n = 6 mice, n = 14 cells). (E) Diastolic depolarization rate (DDR) of AnkB^+/−^ SAN cells is increased upon 50 nM ISO but decreased at 0.1 and 0.5 μM CCH plus 50 nM ISO compared to WT SAN cells (WT: n = 5 mice, n = 15 cells; AnkB^+/−^: n = 6 mice, n = 14 cells). (F) Maximum diastolic potential (MDP) between WT and AnkB^+/−^ SAN cells is not different (WT: n = 5 mice, n = 15 cells; AnkB^+/−^: n = 6 mice, n = 14 cells). Data are presented as mean ± SEM. Data were analyzed using 2-way repeated-measures ANOVA with Benjamini, Krieger, and Yekutieli's 2-stage linear step-up procedure for multiple comparisons (A and B), Mann-Whitney *U* test (C), and linear mixed-effects model with Benjamini-Hochberg procedure for multiple comparisons (D, E, and F); ∗*P* < 0.05, ∗∗*P* < 0.01, ∗∗∗∗*P* < 0.0001. CCH = carbachol; HR = heart rate; ISO = isoproterenol; SAN = sinoatrial node.

To determine whether AnkB^+/−^ SAN cells retain the same CCH-mediated effects on HR, we measured AP firing rates in SAN cells exposed to the identical ISO and CCH concentrations used in excised heart studies. SAN cells from AnkB^+/−^ hearts showed reduced AP firing rates at basal conditions compared with SAN cells isolated from WT hearts (Figure 3D; ∗*P* < 0.05). Coapplication of CCH and 50 nM ISO significantly reduced the AP firing rates at 0.1 μM and showed a trending reduction at 0.5 μM CCH (Figure 3D; ∗*P* < 0.05, ∗∗*P* < 0.01, ∗∗∗∗*P* < 0.0001). Interestingly, 1, 5, and 10 μM CCH completely arrested AP firing. DDR was relatively decreased in AnkB^+/−^ SAN cells after coapplication of CCH and 50 nM ISO at 0.1 and 0.5 μM CCH (Figure 3E; ∗*P* < 0.05, ∗∗*P* < 0.01, ∗∗∗∗*P* < 0.001). No changes were noted in MDP between WT and AnkB^+/−^ SAN cells at 50 nM ISO and 0.1 μM CCH (Figure 3F).

### *I*_Ca,L_ but not *I*_f_ is reduced in AnkB^+/−^ SAN

In SAN, *I*_Ca,L_ plays a major role in generating diastolic depolarization^29^ and regulates ryanodine receptor–dependent Ca^2+^ release during the late phase of diastolic depolarization.^29^, ^30^, ^31^ Thus, *I*_Ca,L_ represents a logical mechanism for reduced HR in AnkB^+/−^ SAN. We investigated WT and AnkB^+/−^ SAN cells for alterations in *I*_Ca,L_ using voltage-clamp protocols designed to minimize T-type Ca^2+^ channel currents. Notably, we observed a significant reduction in *I*_Ca,L_ density in AnkB^+/−^ single SAN cells (Figures 4A and 4B; ∗∗∗*P* < 0.001, ∗∗∗∗*P* < 0.0001), consistent with prior observations in atria^5^ and SAN cells.^4^ Of note, previous work highlighted a role of AnkB in the regulation of Ca_v_1.3 membrane targeting and function in atrial myocytes^5^ and SAN cells.^4^ We then assessed whether SR Ca^2+^ is altered by sympathetic stimulation in AnkB^+/−^ SAN cells at baseline, +ISO, and +ISO/CCH. SAN SR Ca^2+^ content was measured by integrating *I*_NCX_ in response to a “spritz” of caffeine. Notably, SR Ca^2+^ content was significantly lower in AnkB^+/−^ SAN cells than WT cells at baseline (Supplemental Figure 4; ∗*P* < 0.05). Application of 50 nM ISO resulted in equivalent SR Ca^2+^ content between WT and AnkB^+/−^ SAN cells, suggesting a higher net increase of SR Ca^2+^ in AnkB^+/−^ vs WT SAN cells. Further, we found that addition of CCH reduced SR Ca^2+^ content to equivalent minimal levels in WT and AnkB^+/−^ SAN cells (Supplemental Figure 4B).

**Figure 4:**
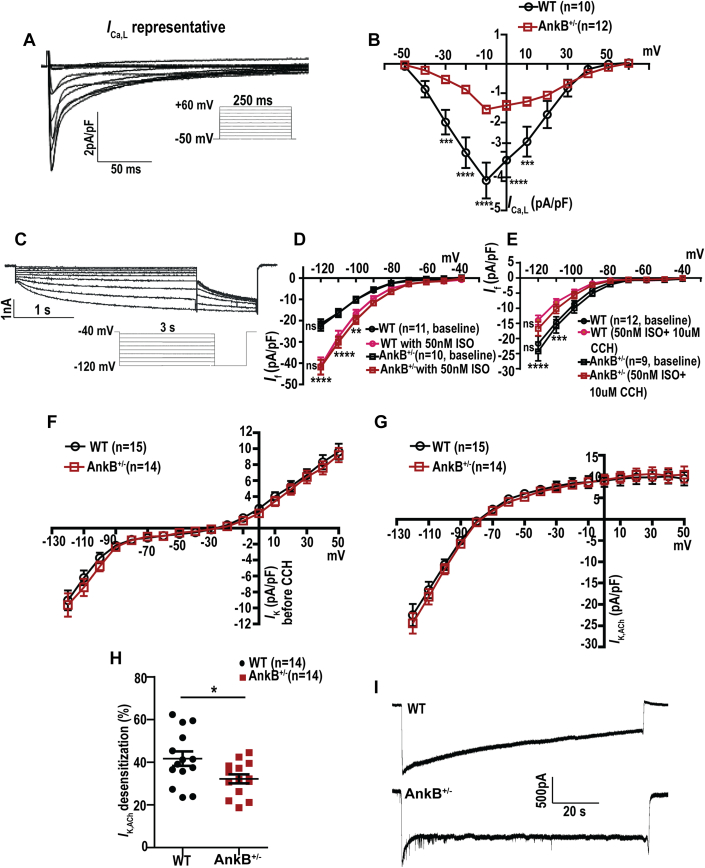
AnkB Deficiency Is Independent of *I*_f_ but Alters *I*_K,ACh_ Desensitization (A and B) Representative recordings of *I*_Ca,L_ and current-voltage relationship showing AnkB^+/−^ SAN cells with a significant reduction in *I*_Ca,L_ density compared to WT SAN cells (WT: n = 6 mice, n = 10 cells; AnkB^+/−^: n = 7 mice, n = 12 cells). (C to E) No change in *I*_f_ density is noted at baseline, as well as following sympathetic (WT: n = 6 mice, n = 11 cells; WT ISO: n = 6 mice, n = 11 cells; AnkB^+/−^: n = 4 mice, n = 10 cells; AnkB^+/−^ ISO: n = 4 mice, n = 10 cells) and sympathetic/parasympathetic activation (ISO + CCH) (WT: n = 7 mice, n = 12 cells; WT [ISO + CCH]: n = 7 mice, n = 12 cells; AnkB^+/−^: n = 6 mice, n = 9 cells; AnkB^+/−^ [ISO + CCH]: n = 6 mice, n = 9 cells) between WT and AnkB^+/−^ SAN cells. Significance is noted between treated and untreated within the same group. (F) No change in *I*_k_ density is noted between WT and AnkB^+/−^ SAN cells (WT: n = 8 mice, n = 15 cells, AnkB^+/−^: n = 8 mice, n = 14 cells). (G) No change in *I*_K,ACh_ density between WT and AnkB^+/−^ SAN cells (WT: n = 8 mice, n = 15 cells; AnkB^+/−^: n = 8 mice, n = 14 cells). (H) Quantitative analysis showing that time-dependent decline of *I*_K,ACh_ is significantly reduced in AnkB^+/−^ SAN cells compared to WT (WT: n = 8 mice, n = 14 cells; AnkB^+/−^: n = 7 mice, n = 14 cells). (I) Representative recording of *I*_K,ACh_ desensitization. Data are presented as mean ± SEM. Data were analyzed using linear mixed-effects model with Benjamini-Hochberg procedure for multiple comparisons (B, F, and H) and generalized linear mixed-effects model with Benjamini-Hochberg procedure for multiple comparisons (D, E, and G); ∗*P* < 0.05, ∗∗*P* < 0.01, ∗∗∗*P* < 0.001, ∗∗∗∗*P* < 0.0001. Abbreviations as in Figure 3.

HCN4-encoded *I*_f_ is critical for regulating SAN spontaneous activity and cardiac rate control.^28^^,^^32^ Further, *I*_f_ is controlled by intracellular cAMP, and therefore activated by β_1_-adrenergic stimulation and inhibited by M_2_R stimulation. We therefore tested *I*_f_ current at baseline, as well as after sympathetic and sympathetic/parasympathetic activation (ISO ± CCH) in WT and AnkB^+/−^ SAN cells. No significant difference in *I*_f_ density was observed between WT and AnkB^+/−^ SAN cells at baseline (Figures 4C to 4E). Further, no significant differences in *I*_f_ density were noted in both WT and AnkB^+/−^ SAN cells after sympathetic and sympathetic/parasympathetic activation (Figures 4C to 4E). Thus, AnkB SAN dysfunction is independent of *I*_f_.

### AnkB deficiency alters *I*_K,ACh_ desensitization

Parasympathetic regulation of SAN activity involves the release of acetylcholine from post-ganglionic cholinergic neurons, where it binds and activates muscarinic M_2_ receptors of the SAN. This activation promotes the separation of the G_βγ_ subunits that bind and open the G protein–gated inwardly rectifying K^+^ channel, K_ACh_. This results in cellular hyperpolarization and HR slowing.^15^^,^^33^ No changes were noted in *I*_K_ (basal) density (before CCH) in both WT and AnkB^+/−^ SAN cells (Figure 4F). We then measured *I*_K,ACh_ density in isolated WT and AnkB^+/−^ SAN cells. Whereas the peak *I*_K,ACh_ or I-V relationships were similar between the groups (Figure 4G), the time-dependent decline of *I*_K,ACh_ was significantly reduced in AnkB^+/−^ SAN cells compared with WT (Figures 4H to 4I; ∗*P* < 0.05), suggesting that enhanced CCH signaling in the SAN of AnkB^+/−^ mice is because of the result of altered *I*_K,ACh_ desensitization. Previous work has shown that regulators of G protein signaling (RGS proteins) are modulators of parasympathetic activation in the heart. In fact, RGS6 and RGS4 knockout mice showed a significant reduction in the extent of *I*_K,ACh_ desensitization.^23^^,^^34^ However, we observed no significant changes in RGS6 and RGS4 protein expression in AnkB^+/−^ atrial lysates compared to control WT atrial lysates (Supplemental Figure 5).

### Ankyrin-B associates with GIRK1 and GIRK4 subunits of the K_ACh_ channel

The K_ACh_ channel is a heterotetramer of 2 G protein–coupled inwardly-rectifying K1 (GIRK1, Kir3.1) subunits and 2 GIRK4 (Kir3.4) subunits.^8^ AnkB^+/−^ atrial lysates displayed reduced ankyrin-B protein expression compared to control WT atrial lysates (Figures 5A and 5B; ∗∗*P* < 0.01). As ankyrins have never been linked with GIRK regulation, we were surprised to detect a decrease in atrial GIRK1 expression in AnkB^+/−^ hearts compared to WT control mice (Figures 5C and 5D; ∗*P* < 0.05). Interestingly, GIRK4 atrial expression was not different between AnkB^+/−^ and control lysates (Figures 5E and 5F). The significant reduction in GIRK1 levels suggests a possible role for AnkB in the membrane abundance of these subunits in vivo and the formation of a heterotetrameric channel. We also evaluated the ability of AnkB to associate with GIRK1 and GIRK4 in mouse heart and atrial tissues. Co-immunoprecipitation experiments demonstrate that AnkB co-immunoprecipitates with GIRK1 and GIRK4 from total heart and atrial lysates (Figures 5G to 5J). We observed no interaction between anti-AnkB and control Ig immunoprecipitates from any lysate. Alternatively, GIRK1 and GIRK4 co-immunoprecipitate with AnkB from total heart and atrial lysates (Supplemental Figure 6). Collectively, these data suggest an in vivo interaction between AnkB and K_ACh_ subunits in the heart.

**Figure 5:**
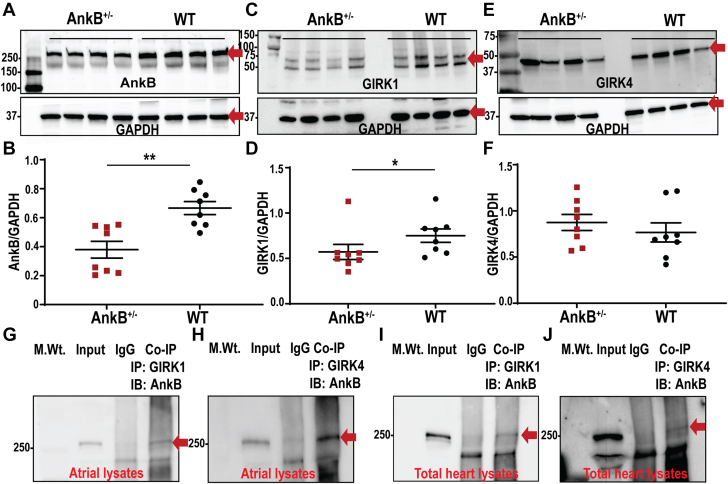
AnkB Associates With K_ACh_ Channel Subunits in Cardiac Lysates Atrial lysates from AnkB^+/−^ and wild type (WT) mice were evaluated for GIRK1 and GIRK4 expression. (A and B) Immunoblots and quantitative analysis of AnkB expression normalized to GAPDH in atrial lysates (n = 8 mice/group). (C and D) Immunoblots and quantitative analysis of GIRK1 expression normalized to GAPDH in atrial lysates (n = 8 mice/group). (E and F) Immunoblots and quantitative analysis of GIRK4 expression normalized to GAPDH in atrial lysates (n = 8 mice/group). (G to J) Anti-GIRK1 Ig and anti-GIRK4 Ig co-immunoprecipitate ankyrin-B from whole heart and atrial lysates. There is no interaction between anti-AnkB and control IgG immunoprecipitates from any lysate. Co-immunoprecipitation (Co-IP) experiments were repeated 3 times. Data are presented as mean ± SEM. Data were analyzed using Mann-Whitney U test (B and D) and unpaired Student's t-test (F); ∗*P* < 0.05, ∗∗*P* < 0.01.

### Ank2-cKO mice display defects in atrial GIRK1 membrane localization

Dysfunction of *I*_K,ACh_ in humans and animal models results in abnormal heart regulation and arrhythmia.^12^^,^^35^, ^36^, ^37^ In fact, aberrant regulation of *I*_K,ACh_ is also a hallmark of atrial arrhythmia and a major area for the design of therapies.^38^^,^^39^ *I*_K,ACh_ is tightly regulated by the assembly and targeting of channel heterotetramers comprised of 2 GIRK1 and 2 GIRK4 subunits.^11^^,^^13^^,^^40^ We then evaluated the localization of GIRK1 and GIRK4 at the cell membrane of atrial myocytes in cryoslices of *Ank2*-cKO and control floxed mice. While control floxed myocytes demonstrated diffuse and membrane-associated GIRK1 and GIRK4 expression (Figures 6A and 6C), *Ank2*-cKO atrial myocytes demonstrated a significant reduction in membrane-associated GIRK1 (Figure 6B; ∗∗∗*P* < 0.001). Notably, GIRK1 localization was reduced at the membrane in the atrial myocytes of *Ank2*-cKO cryosections, with some detectable GIRK1 signal localized intracellularly. Of note, GIRK4 membrane localization was not changed at the membrane of control and *Ank2*-cKO atrial cryosections (Figure 6D).

**Figure 6:**
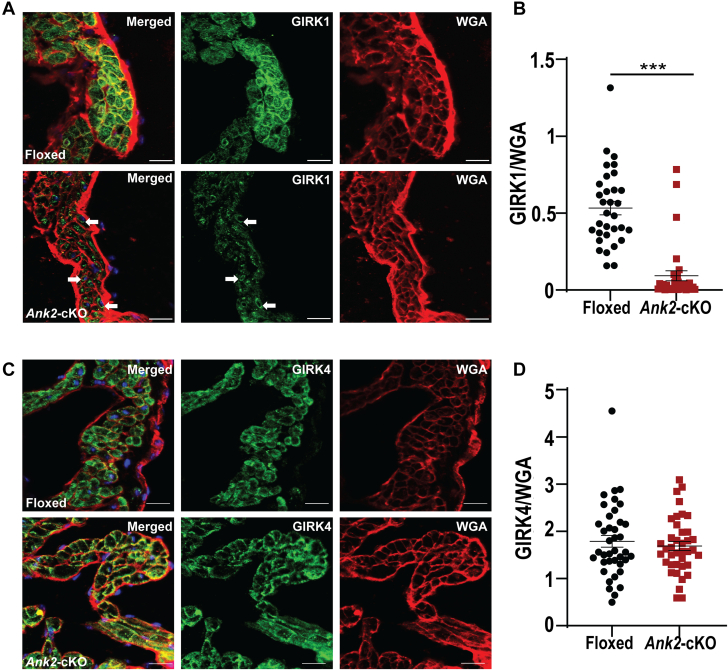
Ank2-cKO Mice Display Changes in Atrial GIRK1 Localization and Expression Without Changes in Atrial GIRK4 Localization and Expression (A and B) Representative atrial cryosection images and quantification from floxed and *Ank2*-cKO mice stained with GIRK1 (green), Wheat Germ Agglutinin (WGA; red) and overlap including DAPI nuclear stain (blue). Scale bars equal 20 μm. Images are representative of n = 3 mice/group, n = 5 to 6 sections/group. (C and D) Representative atrial cryosection images and quantification from floxed and *Ank2*-cKO mice stained with GIRK4 (green), WGA (red) and overlap including DAPI nuclear stain (blue). Scale bars equal 20 μm. Images are representative of n = 3 mice/group and n = 5 to 6 sections/group. Data are presented as mean ± SEM. Data were analyzed using generalized linear mixed-effects model (B, D); ∗∗∗*P* < 0.001.

GIRK1 and GIRK4 subunits must form heterotetramers for normal/native *I*_K,ACh_ regulation.^11^, ^12^, ^13^ For nearly 2 decades, a “chaperone” for the post-ER assembly and membrane targeting/localization of GIRK1/4 heterotetramers has been proposed.^13^^,^^41^ Our biotinylation studies (Supplemental Figure 7) show no significant changes in membrane GIRK1 (biotin-bound) to total GIRK1 (input) protein ratio in *Ank2*-cKO atrial lysates compared to control atrial lysates, suggesting a role of AnkB in the abundance of native GIRK heterotetramers.

## Discussion

The automaticity of the human SAN is orchestrated by the integration of the opposing activities of sympathetic and parasympathetic systems. Impaired modulation of the SAN by the autonomic nervous system signaling has been associated with human heart failure^42^ and arrhythmia.^43^^,^^44^ SAN dysfunction also results in increasing pacemaker implantation by more than 100% (102%) compared with all other indications for implantation.^45^^,^^46^ However, whether uncoordinated modulation of SAN to sympathetic and parasympathetic signaling can be pathogenic is still matter of debate. Therefore, elucidating the regulators of SAN response to autonomic stimulation is critical for human cardiovascular health.

SAN pacemaker cells have intrinsic automaticity via 2 mechanisms: the membrane clock and the Ca^2+^ clock. These 2 mechanisms work in tandem (denoted the “coupled clock” mechanism) to facilitate rhythmic action potential firing, while the G protein–coupled receptors allow modulation of the rate.^47^ The parasympathetic nervous system activates the muscarinic receptors from acetylcholine release by the vagal nerve.^48^ Sporadic and irregular parasympathetic activity has long been associated with sinus arrhythmia and sinus node dysfunction.^49^

This study highlights the coexistence of the enhancement of cardiac sympathetic and parasympathetic signaling in animal disease models of AnkB deficiency. We demonstrate that AnkB deficiency causes a reduction in baseline HR but an increased HR response to the sympathetic and parasympathetic agonists in vivo, in isolated hearts and SAN cells of AnkB^+/−^ mice. We also noted an increase in all HRV parameters at baseline in AnkB^+/−^ mice compared to controls. Reduced rates and increased HRV in AnkB^+/−^ mice were more obvious at baseline versus post-stimulation, suggesting an exaggerated parasympathetic control of HR in AnkB^+/−^ mice. Our data also show a significant reduction in *I*_Ca,L_ density in AnkB^+/−^ SAN cells. This finding could explain decreased basal pacemaker activity phenotypes in *ANK2* patients and AnkB^+/−^ mice.

*I*_K,ACh_ is a critical mechanism in the neurohumoral modulation of SAN pacemaker activity and HR regulation during the fight-or-flight response.^15^ In the SAN, *I*_K,ACh_ activation negatively regulates spontaneous AP firing rate by hyperpolarizing the MDP, slowing down the diastolic depolarization rate and HR.^15^^,^^50^^,^^51^ Previous work using GIRK4^−/−^ mice has demonstrated diminished cholinergic regulation of SAN pacemaking activity and a reduction in the speed of recovery of resting HR after physiological events that mimic the fight-or-flight response.^15^ GIRK4 is therefore necessary for fast regulation of HR in response to vagal stimulation and at rest.^52^ Therefore, we hypothesized a novel potential role for AnkB in the regulation of GIRK/*I*_K,ACh_ and autonomic regulation.

GIRK subunits confer different trafficking patterns on K_ACh_ channels. GIRK1 is unique in that it is unable to form functional channels by itself^8^^,^^53^^,^^54^ but requires combination with another GIRK (GIRK2 or GIRK4), which can, by themselves, form functional homotetramers. Moreover, GIRK4 has been demonstrated to induce the glycosylated mature form of GIRK1, suggesting that association with GIRK4 during, or shortly after, subunit synthesis is necessary for GIRK1 processing and localization.^13^ Interestingly, while most of the work regarding the trafficking of K_ACh_ channels has been performed in neurons, very little has been shown in the heart. In this study, we demonstrate a role of ankyrin-B in the abundance and membrane localization of native cardiac GIRK channels.

Reduced *I*_K,ACh_ desensitization can be attributed to several mechanisms, including impaired RGS protein function because of reduced expression or activity (eg, RGS4 and RGS6),^23^^,^^34^ channel-level alterations such as changes in subunit stoichiometry or post-translational modifications,^55^ reduced receptor desensitization,^56^^,^^57^ and altered Ca^2+^-dependent feedback mechanisms, among others.^48^ Our study suggests that an AnkB-dependent pathway is involved in the expression and localization of GIRK subunits. Loss of AnkB, as seen in AnkB^+/−^ and *Ank2*-cKO mice, results in a reduction in GIRK1 expression in hearts, loss of proper membrane localization of these subunits, and alterations in SAN cell electrophysiology. One potential explanation for the changes noted in GIRK1 expression with unchanged peak *I*_K,ACh_ density and altered *I*_K,ACh_ desensitization is the ability of GIRK4 to form homotetramers.^8^^,^^11^^,^^58^ Cardiac K_ACh_ is tightly regulated by the assembly and targeting of channel heterotetramers comprising 2 GIRK1 and 2 GIRK4 subunits.^8^^,^^9^ K_ACh_ can exist either as a heterotetramer comprising 2 GIRK1 and 2 GIRK4^8^ or as a homotetramer of 4 GIRK4.^59^ However, the expression of only GIRK4 in atrial myocytes resulted in loss of acute desensitization and changes in K_ACh_ properties.^11^^,^^13^ Therefore, GIRK1 and 4 subunits must form heterotetramers to maintain proper/native *I*_K,ACh_ regulation.^11^, ^12^, ^13^ Thus, this study confirms AnkB as a potential regulator of SAN response to autonomic nerve stimulation by regulating the expression and localization of both GIRK subunits in the atria and SAN, and it provides novel insight into the underlying mechanism of the HR variability experienced in models of AnkB deficiency.

Although AnkB and GIRK4 co-immunoprecipitate, previously reported GIRK4^−/−^ mice exhibited normal resting mean HR but reduced HR variability, consistent with impaired parasympathetic regulation due to loss of *I*_K,ACh_. In contrast, AnkB^+/−^ mice displayed a distinct phenotype. The difference is likely attributable to the abnormal Ca^2+^ handling associated with AnkB deficiency. Furthermore, the altered *I*_K,ACh_ desensitization observed in AnkB-deficient mice is likely due to reduced expression and localization of *I*_K,ACh_ heterotetramer subunits. These findings provide further mechanistic insight into abnormal HR regulation in AnkB deficiency. The slow recovery from sympathetic and parasympathetic stimulation could result in periods of extreme high and low HRs, thereby increasing the window of vulnerability for arrhythmia initiation. Identifying ankyrin-B as an upstream regulator of *I*_K,ACh_ is critical, as ankyrin-B reduction has been found in multiple forms of congenital^6^ and acquired heart disease, including heart failure^60^ and ischemic heart disease.^61^^,^^62^

## Funding Support and Author Disclosures

The authors are supported by National Institutes of Health grants HL157684 (to Dr Nassal), HL171689, HL156652 and HL165751 (to Dr Hund), HL135754 (to Dr P. Mohler), and HL146969 (to Dr El Refaey), as well as a grant from the Ohio State Frick Center for Heart Failure and Arrhythmia, the JB Cardiovascular Fellowship, and the Fondation Leducq (TNE FANTASY 19CV03) (to Drs P. Mohler. and Mangoni). The content is solely the responsibility of the authors and does not necessarily represent the official views of the National Institutes of Health. The authors have reported that they have no relationships relevant to the contents of this paper to disclose.

Perspectives **COMPETENCY IN MEDICAL KNOWLEDGE:** Ankyrin-B is a key cytoskeletal-associated protein that plays a crucial role in the targeting and localization of major ion channels in the heart. This study enhances our understanding of the role of AnkB in mediating the balance between sympathetic and parasympathetic modulation of the SAN. Bradycardia and elevated baseline HRV observed in AnkB-deficient models suggest heightened parasympathetic influence. The study provides further mechanistic insight into SAN dysfunction by emphasizing the role of AnkB in the membrane abundance of *I*_K,ACh_ channel subunits and Ca^2+^ regulation.

**TRANSLATIONAL OUTLOOK:** Our study identifies AnkB as a novel upstream regulator of *I*_K,ACh_ and GIRK channel subunit expression and membrane localization in cardiac myocytes. Ankyrin-B reduction/deficiency has been found in multiple forms of congenital and acquired heart disease. Targeting this pathway may provide novel strategies for the treatment of autonomic cardiac dysregulation and SAN dysfunction. Modulation of GIRK1/GIRK4 heterotetramer localization and stabilization may represent a novel therapeutic avenue for patients with autonomic imbalance and SAN dysfunction.
