# Supplementary material for: Dysfunction in Ankyrin-B Disrupts Heart Rate Regulation by Altering the Membrane Abundance of GIRK Channels
Source: JACC Basic Transl Sci. 2026 Aug 11;11(9):101647. doi: 10.1016/j.jacbts.2026.101647 (PMC13482378; doi:10.1016/j.jacbts.2026.101647)
Supplement: Supplementary file 1 [file mmc1.docx]

**Supplemental Appendix**

**Dysfunction in ankyrin-B disrupts heart rate regulation by altering the membrane abundance of GIRK channels**

Jingdong Li, MD, PhD^1, #^, Crystal F. Kline, PhD^1, #^, Omer Cavus, MD^1,2^, John T. Bobik, BS^1,2^, Nipun Malhotra, MSc^1,2^, Jagun Raghavan, BS^1,2^ , Sarah S. Takenaka, BS^1,2^, Junie Kwak, BS^1,2^, Ryan J. Cordeiro, BS^1,2^, Eleanor J. Mohler^1^, Emma Dudley, MD^1,3^, Xianyao Xu, MSc^1^, Drew M. Nassal, PhD^1,3^, Yuejin Wu, PhD^4^, Thomas J. Hund, PhD^1,5,6^, Matteo E. Mangoni, PhD^7,8^, Peter J. Mohler, PhD^1,3,6^ and Mona El Refaey, PhD^1,2, *^

^1^ The Frick Center for Heart Failure and Arrhythmia, Dorothy M. Davis Heart and Lung Research Institute, The Ohio State University, Columbus, OH, 43210.

Departments of ^2^ Surgery, Division of Cardiac Surgery, ^3^ Physiology and Cell Biology, The Ohio State University College of Medicine and Wexner Medical Center, Columbus, OH 43210.

^4^ Department of Medicine, Johns Hopkins University, Baltimore, MD, 21205.

^5^ Department of Biomedical Engineering, College of Engineering, The Ohio State University, Columbus, OH, 43210.

^6^ Department of Internal Medicine, Division of Cardiovascular Medicine, The Ohio State University College of Medicine and Wexner Medical Center, Columbus, OH, 43210.

^7^ Institut de Génomique Fonctionnelle, Université de Montpellier, CNRS, INSERM, Montpellier, France

^8^ Laboratories of Excellence, Ion Channel Science and Therapeutics, 06560 Valbonne, France

^#^ Equal contribution

Short title: Role of AnkB in the regulation of GIRK channels

*Address Correspondence to:

Mona El Refaey, PhD, MS

Department of Surgery/Division of Cardiac Surgery

The Dorothy M. Davis Heart and Lung Research Institute

The Ohio State University College of Medicine and Wexner Medical Center

333 West 10^th^ Avenue, Columbus, OH, 43210

[Mona.elrefaey@osumc.edu](mailto:Mona.elrefaey@osumc.edu)

Tel: 614-366-2748

**Conflict of Interest Statement**

The authors have no conflict of interest.

**Supplemental FIGURE 1**

***Heart rate variability (HRV) parameters are altered in AnkB deficiency.* (Aa-Ah)** Conscious ECG recordings from AnkB^+/-^ and control mice at baseline without stimulation denote an increase in Standard Deviation of RR intervals (SDRR), the percentage of successive RR interval pairs that differ by more than 6 milliseconds (pNNper6ms), short-term variability of heart rate (SD1), long-term variability in RR intervals (SD2), Low Frequency (LF) component of HRV (0.04 to 0.15 Hz) and High-Frequency component (HF) of HRV (0.15 to 0.40 Hz). **(Ba-Bh)** Injection of isoproterenol (1 mg/kg) abolishes the differences between mice in AnkB^+/-^ and control mice in all HRV parameters. **(Ca-Ch)** Injection of carbachol (0.15 mg/kg) shows no significant changes in all HRV parameters across the groups (WT N=7 mice, AnkB^+/-^ N=7 mice). N.B. Log values of LF and HF segments were added to normalize skewed HRV data. Data are presented using mean ± SEM. Data were analyzed using unpaired Student’s t-test (A; B: d, g, h; C: a, d, g, h) and Mann-Whitney U test (B: a, b, c, e, f; C: b, c, e, f), *p<0.05, **p<0.01.

**Supplemental FIGURE 2**

***AnkB deficiency does not significantly change the APD duration of SAN cells.*** (**A**) APD90, APD75 and APD50 at baseline from SAN cells of WT and AnkB^+/-^ mice (WT N=5 mice, n=13 cells; AnkB^+/-^ N=9 mice, n=14 cells). (**B**) APD90, APD75 and APD50 at ISO 50 nM from SAN cells of WT and AnkB^+/-^ mice (WT N=5 mice, n=13 cells; AnkB^+/-^ N=9 mice, n=14 cells). Data are presented using mean ± SEM. Data were analyzed using linear mixed effects model (A: APD90, APD 50; B) and generalized linear mixed effects model (A: APD75).

**Supplemental FIGURE 3**

***No changes of SAN recovery time or AV node conduction in AnkB deficiency.* (A)** QT_90_, QT_75_ and QT_50_ from surface ECGs recordings are significantly prolonged in AnkB^+/-^ mice compared with WT controls (WT N=20 mice, AnkB^+/-^ N=22 mice). **(B)** Same parameters in Langendorff-perfused WT and AnkB^+/-^ hearts are significantly increased in AnkB^+/-^ hearts compared to WT hearts (WT N=20 mice, AnkB^+/-^ N=22 mice). **(C)** WT and AnkB^+/-^ hearts show no difference in corrected sinus node recovery time (SNRTc), atrioventricular effective refractory periods (AV-ERP), AV nodal Wenckebach cycle length (AV-WBCL), and AV 2:1 conduction cycle length (AV 2:1). Left ventricular effective refractory periods (LV-ERP) are significantly different between WT and AnkB^+/^ hearts (WT N=15 mice, AnkB^+/-^ N=19 mice). Data are presented using mean ± SEM. Data were analyzed using unpaired Student’s t-test (A: QT90; B: QT90 and QT75; C: SNRTc, AVWBCL, AV2:1, LVERP) and Mann-Whitney U test (A: QT75 and QT50; B: QT50; C: AVERP), *p<0.05, **p<0.01, ***p<0.001.

**Supplemental FIGURE 4**

***SR Ca^2+^ content is reduced in in AnkB^+/-^ SAN cells at baseline but increased upon ISO challenge and decreased by ISO plus CCH application****.* **(A)** Representative *I*_NCX_ recordings from WT and AnkB^+/-^ SAN cells in response to a caffeine spritz. SR Ca^2+^ content is calculated from the integral of *I*_NCX_. **(B)** Summary data for SAN SR Ca^2+^ content at baseline, after 50 nM ISO and after 50 nM ISO+10µM CCH. WT Baseline N=2 mice, n=10 cells; AnkB^+/-^ Baseline N=8 mice, n=12 cells; WT+ISO N=3 mice, n=5 cells; AnkB^+/-^+ISO N=3 mice, n=9 cells; WT+ISO+CCH N=3 mice, n=8 cells; AnkB^+/-^ +ISO+CCH N=8 mice, n=15 cells. Data are presented using mean ± SEM. Data were analyzed using linear mixed effects model (Baseline and ISO) and generalized linear mixed effects model (ISO+CCH). *p<0.05.

**Supplemental FIGURE 5**

***No changes are noted in RGS4 and RGS6 protein expression in atrial lysates isolated from AnkB^+/-^ mice.* (A-B)** Immunoblots and quantitative analysis of RGS4 expression normalized to GAPDH in atrial lysates, N=8 mice/group. **(C-D)** Immunoblots and quantitative analysis of RGS6 expression normalized to GAPDH in atrial lysates, N=8 mice/group. Data are presented using mean ± SEM. Data were analyzed using unpaired Student’s t-test (B, D).

**Supplemental Figure 6**

***K_ACh_ channel subunits associate with AnkB in cardiac lysates.* (A-D)** Anti-ankyrin-B Ig immunoprecipitates ankyrin-B from whole heart and atrial lysates. **(E-H)** Anti-ankyrin-B Ig co-immunoprecipitates GIRK1 and GIRK4 from whole heart and atrial lysates. There is no interaction between antibody (AnkB, GIRK1, GIRK4) and control IgG immunoprecipitates from any lysate. Experiments were repeated three times.

**Supplemental Figure 7**

***GIRK1 membrane expression relative to total GIRK1 in atrial lysates isolated from Ank2-cKO mice following in vivo biotinylation.* (A)** Immunoblotting of GIRK1 and biotinylated proteins in *Ank2*-cKO and control atrial lysates. **(B)** No change in membrane (biotin-bound) to total (input) GIRK1 protein ratio in *Ank2*-cKO atrial lysates vs. control atrial lysates, N=4 mice. Data are presented using mean ± SEM. Data were analyzed using unpaired Student’s t-test (B).
