# Supplementary material for: Dysfunction in Ankyrin-B Disrupts Heart Rate Regulation by Altering the Membrane Abundance of GIRK Channels
Source: JACC Basic Transl Sci. 2026 Aug 11;11(9):101647. doi: 10.1016/j.jacbts.2026.101647 (PMC13482378; doi:10.1016/j.jacbts.2026.101647)
Supplement: Supplementary file 1 [file mmc2.pdf]

Unedited Gels

Dysfunction in ankyrin-B disrupts heart rate regulation by altering the membrane abundance of GIRK channels

Jingdong Li, MD, PhD[1, #], Crystal F. Kline, PhD[1, #], Omer Cavus, MD[1,2], John T. Bobik, BS[1,2], Nipun Malhotra, MSc[1,2], Jagun Raghavan, BS[1,2] , Sarah S. Takenaka, BS[1,2], Junie Kwak, BS[1,2], Ryan J. Cordeiro, BS[1,2], Eleanor J. Mohler[1], Emma Dudley, MD[1,3], Xianyao Xu, MSc[1], Drew M. Nassal, PhD[1,3], Yuejin Wu, PhD[4], Thomas J. Hund, PhD[1,5,6], Matteo E. Mangoni, PhD[7,8], Peter J. Mohler, PhD[1,3,6] and Mona El Refaey, PhD[1,2, *]

[1] The Frick Center for Heart Failure and Arrhythmia, Dorothy M. Davis Heart and Lung Research Institute, The Ohio State University, Columbus, OH, 43210.

Departments of [2] Surgery, Division of Cardiac Surgery, [3] Physiology and Cell Biology, The Ohio State University College of Medicine and Wexner Medical Center, Columbus, OH 43210.

[4] Department of Medicine, Johns Hopkins University, Baltimore, MD, 21205.

[5] Department of Biomedical Engineering, College of Engineering, The Ohio State University, Columbus, OH, 43210.

[6] Department of Internal Medicine, Division of Cardiovascular Medicine, The Ohio State University College of Medicine and Wexner Medical Center, Columbus, OH, 43210.

[7] Institut de Génomique Fonctionnelle, Université de Montpellier, CNRS, INSERM, Montpellier, France

[8] Laboratories of Excellence, Ion Channel Science and Therapeutics, 06560 Valbonne, France

[#] Equal contribution

Short title: Role of AnkB in the regulation of GIRK channels

*Address Correspondence to:

Mona El Refaey, PhD, MS

Department of Surgery/Division of Cardiac Surgery

The Dorothy M. Davis Heart and Lung Research Institute

The Ohio State University College of Medicine and Wexner Medical Center

333 West 10th Avenue, Columbus, OH, 43210

Mona.elrefaey@osumc.edu

Tel: 614-366-2748

Conflict of Interest Statement

The authors have no conflict of interest.

AnkB (Figure 5A)

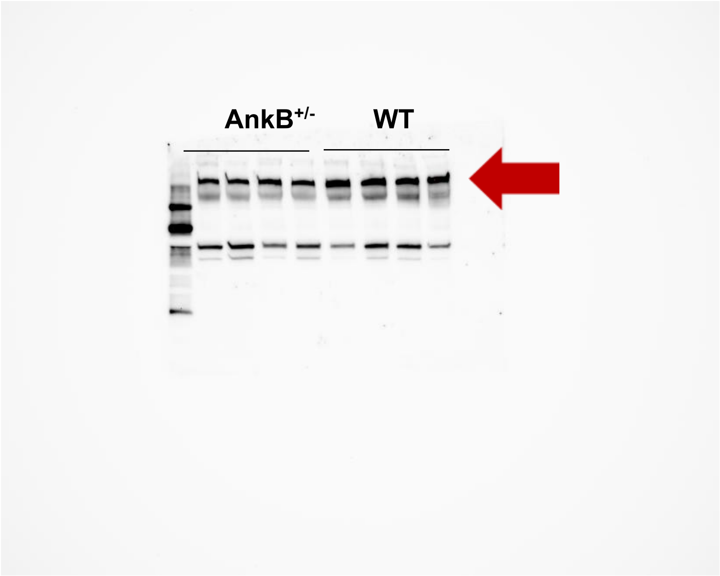

AnkB-GAPDH

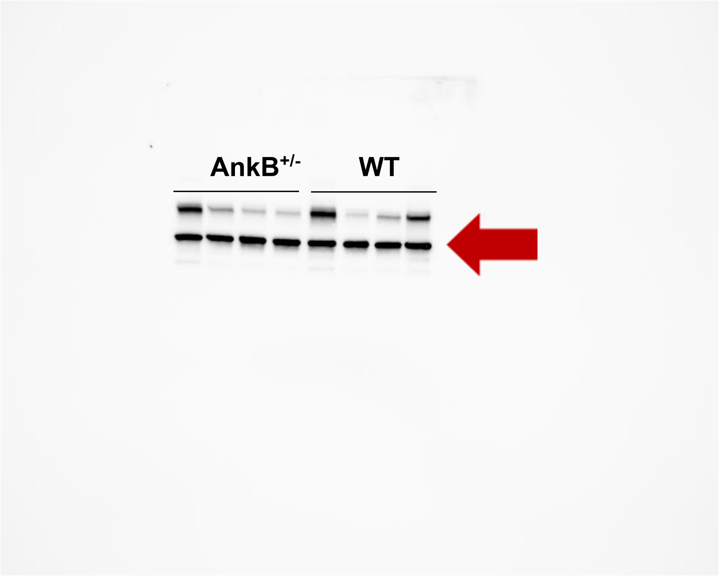

AnkB (Figure 5A)

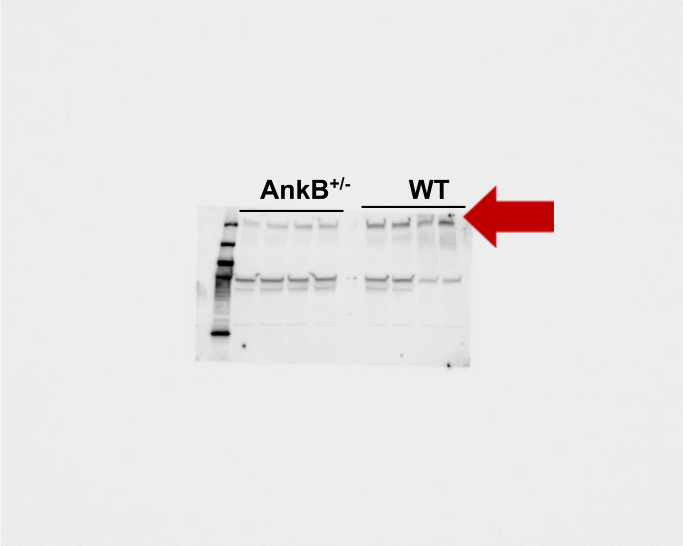

AnkB-GAPDH

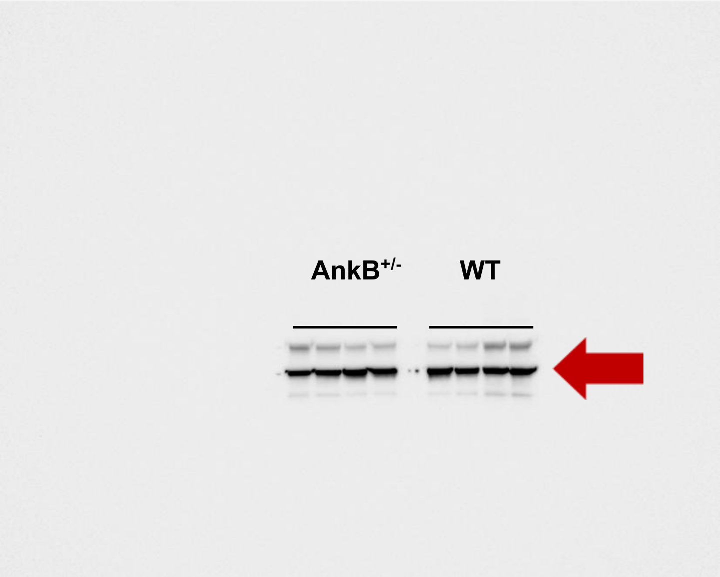

GIRK1 (Figure 5C)

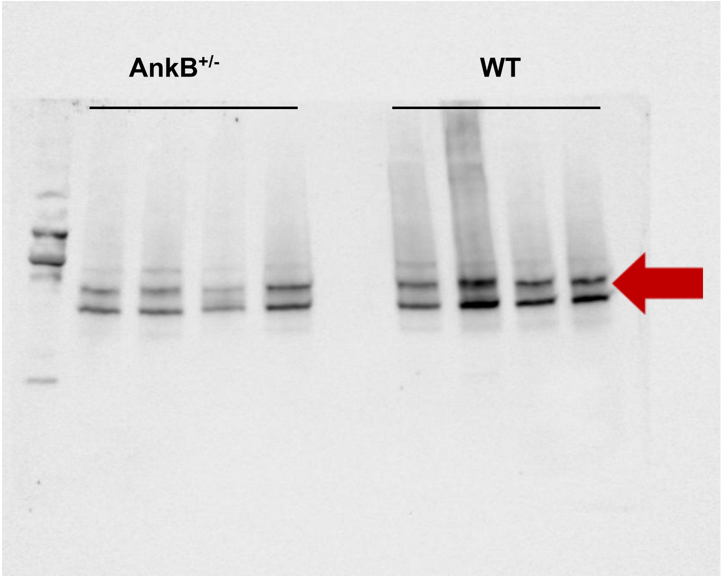

GIRK1-GAPDH

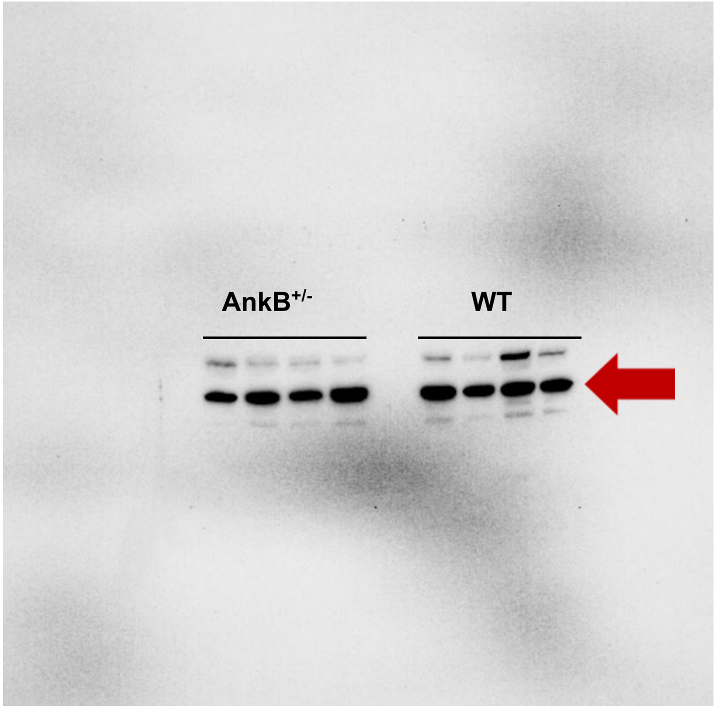

GIRK1 (Figure 5C)

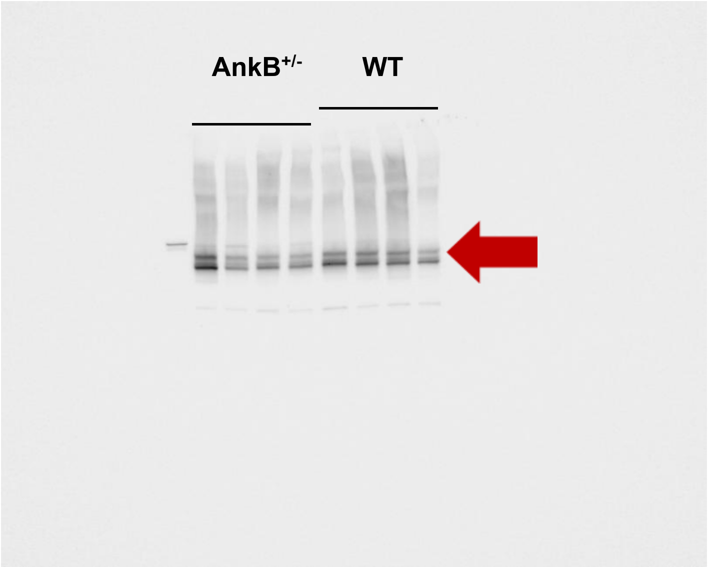

GIRK1-GAPDH

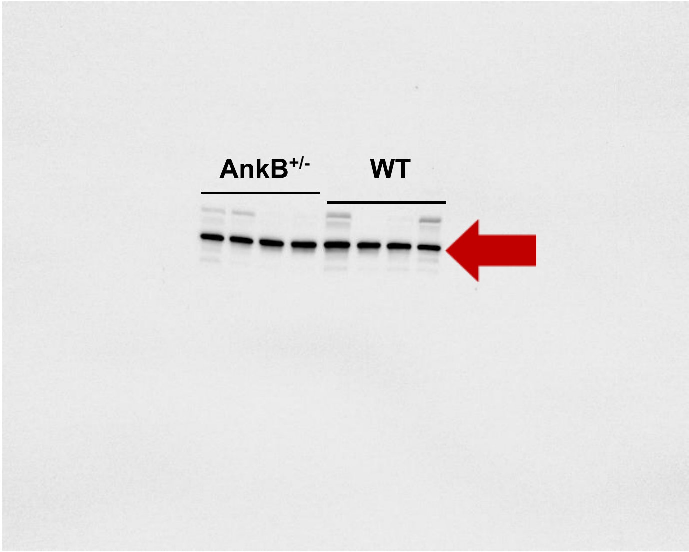

GIRK4 (Figure 5E)

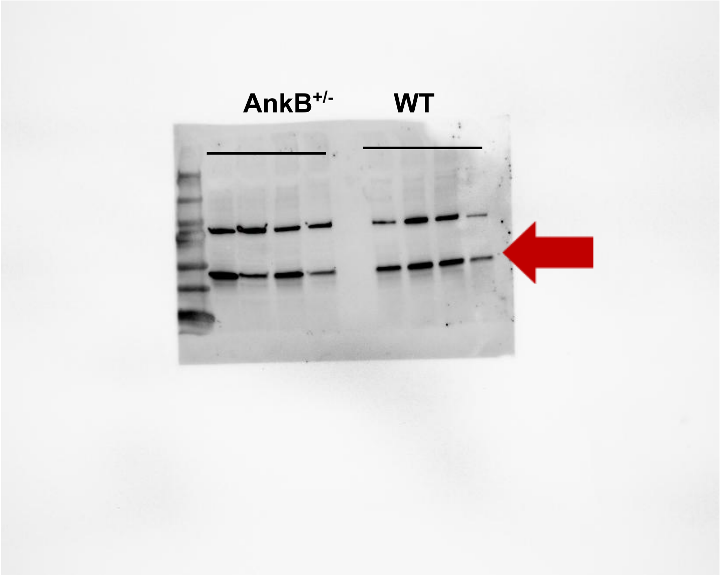

GIRK4-GAPDH

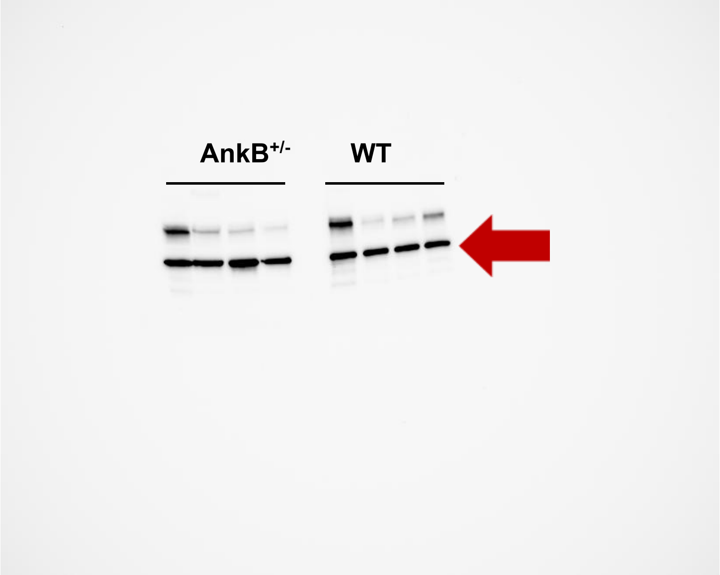

GIRK4 (Figure 5E)

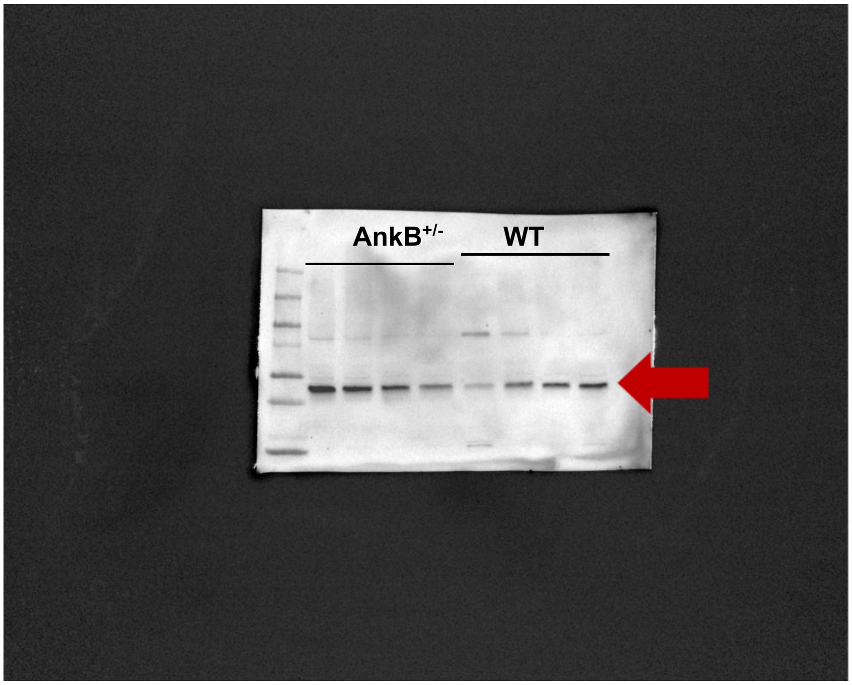

GIRK4-GAPDH

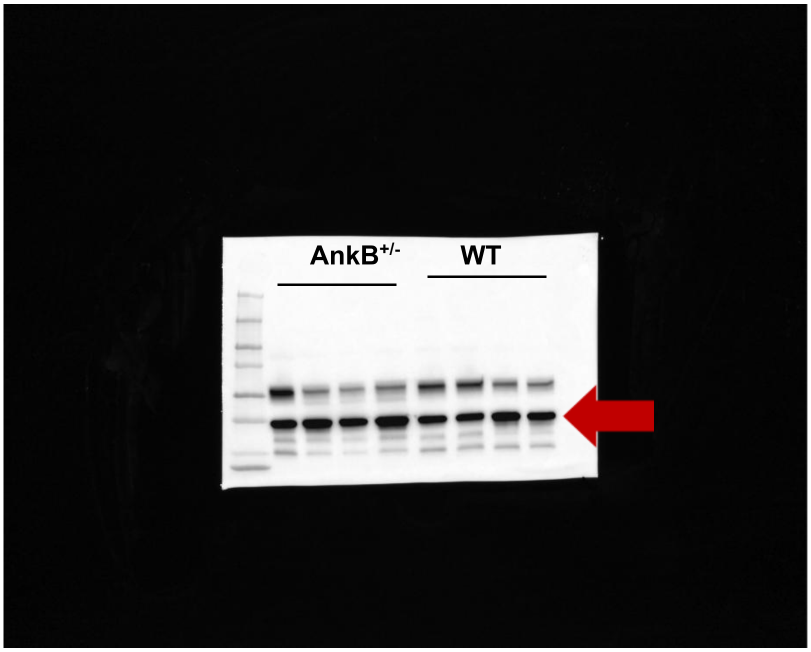

Figure 5G

M.Wt. Input IgG Co-IP

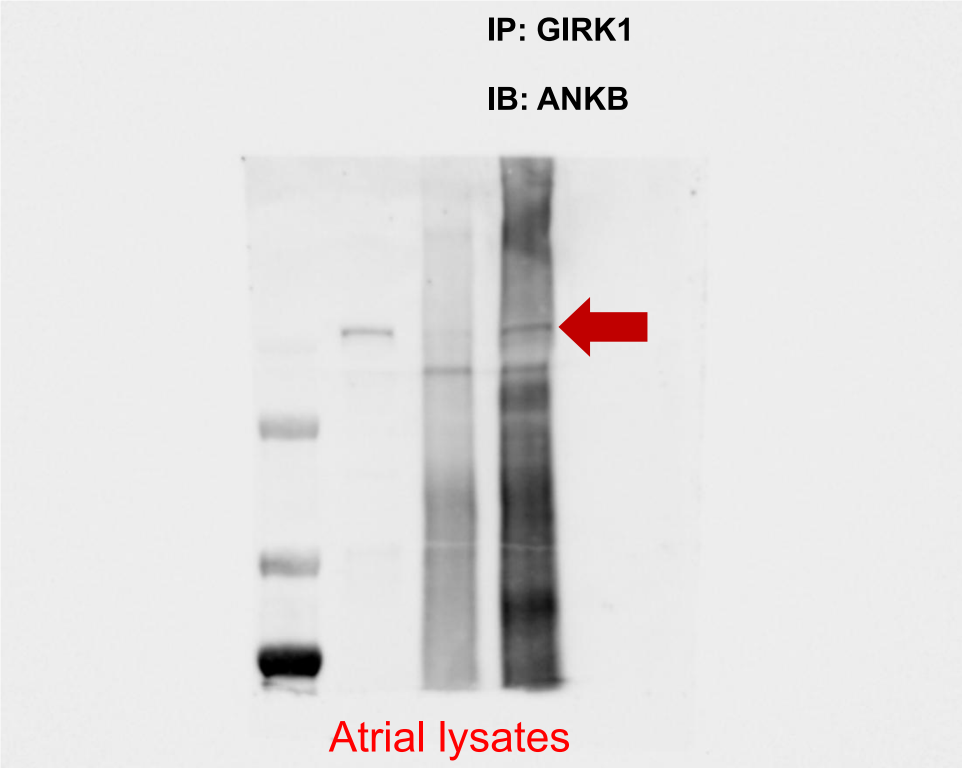

Figure 5H

M.Wt. Input IgG Co-IP

IP: GIRK4

IB: ANKB

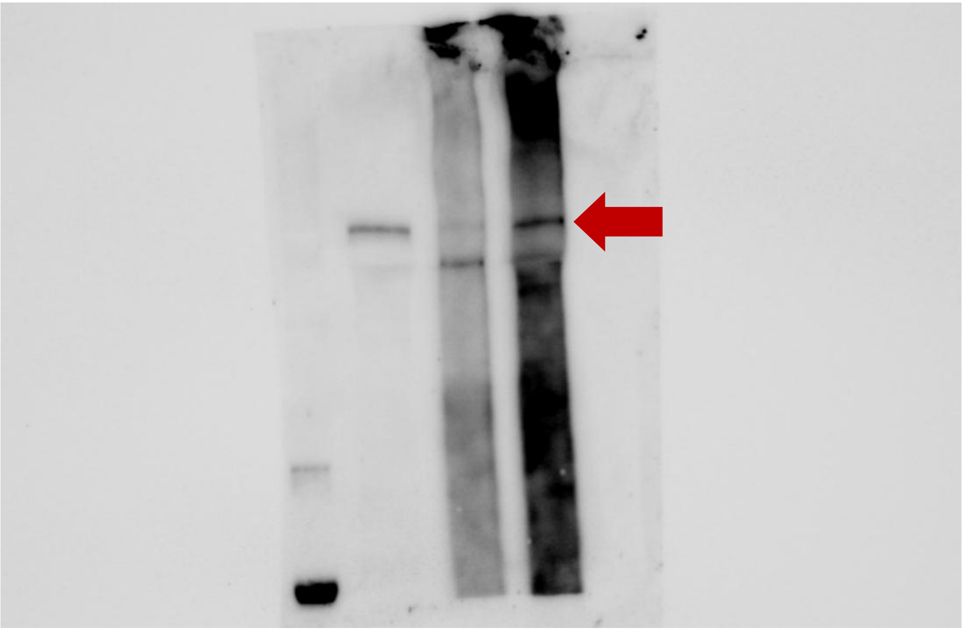

Atrial lysates

Figure 5I

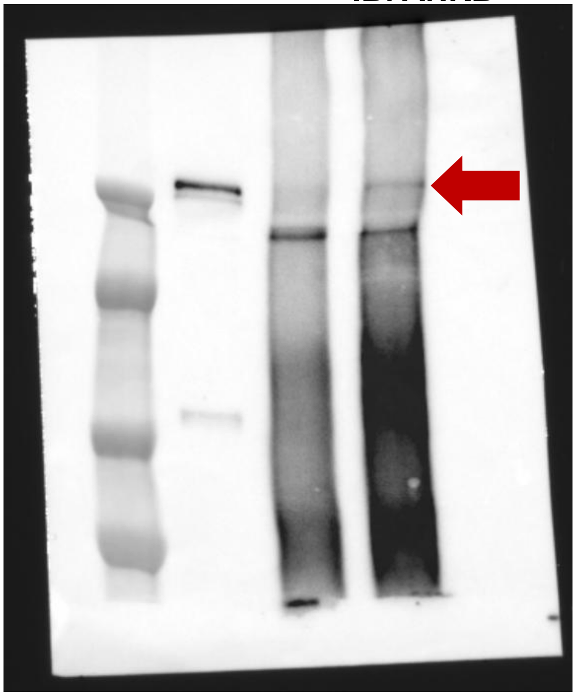

Figure 5J

M.Wt. Input IgG Co-IP

IP: GIRK4

IB: ANKB

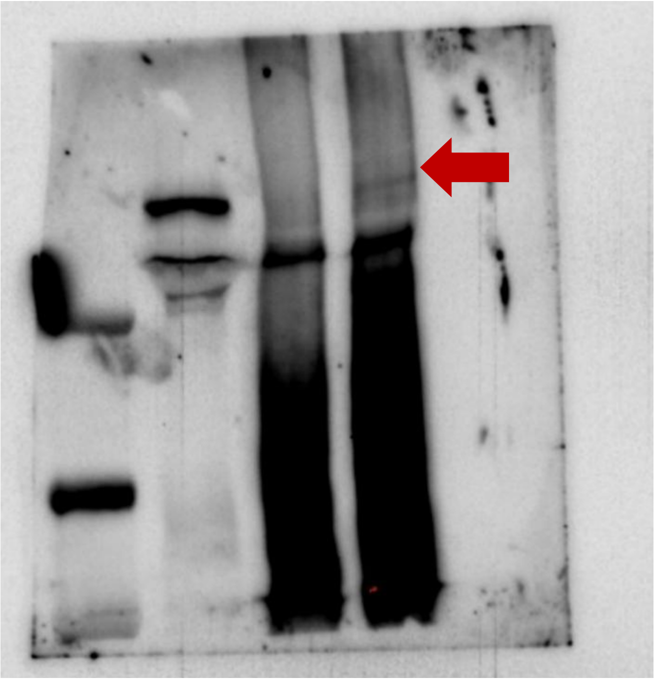

Total heart lysates
